# Targeted up-regulation of Drp1 in dorsal horn attenuates neuropathic pain hypersensitivity by increasing mitochondrial fission

**DOI:** 10.1016/j.redox.2021.102216

**Published:** 2021-12-20

**Authors:** Kun-Long Zhang, Shu-Jiao Li, Xue-Yin Pu, Fei-Fei Wu, Hui Liu, Rui-Qing Wang, Bo-Zhi Liu, Ze Li, Kai-Feng Li, Nian-Song Qian, Yan-Ling Yang, Hua Yuan, Ya-Yun Wang

**Affiliations:** a Specific Lab for Mitochondrial Plasticity Underlying Nervous System Diseases, National Demonstration Center for Experimental Preclinical Medicine Education, The Fourth Military Medical University, Xi'an, 710032, China; b Department of Rehabilitation Medicine, Xi-Jing Hospital, The Fourth Military Medical University, Xi'an, 710032, China; c Department of Human Anatomy, Yan-An University, Yan'an, 716000, China; d Department of Oncology, First Medical Center, The General Hospital of the People's Liberation Army, Beijing, 100000, China; e Department of Liver and Gallbladder Surgery, Xi-Jing Hospital, The Fourth Military Medical University, Xi'an, 710032, China; f State Key Laboratory of Military Stomatology, School of Stomatology, The Fourth Military Medical University, Xi'an, 710032, China

**Keywords:** Drp1, Mitochondria, SNI, Pain, Spinal dorsal horn

## Abstract

Mitochondria play an essential role in pathophysiology of both inflammatory and neuropathic pain (NP), but the mechanisms are not yet clear. Dynamin-related protein 1 (Drp1) is broadly expressed in the central nervous system and plays a role in the induction of mitochondrial fission process. Spared nerve injury (SNI), due to the dysfunction of the neurons within the spinal dorsal horn (SDH), is the most common NP model. We explored the neuroprotective role of Drp1 within SDH in SNI. SNI mice showed pain behavior and anxiety-like behavior, which was associated with elevation of Drp1, as well as increased density of mitochondria in SDH. Ultrastructural analysis showed SNI induced damaged mitochondria into smaller perimeter and area, tending to be circular. Characteristics of vacuole in the mitochondria further showed SNI induced the increased number of vacuole, widened vac-perimeter and vac-area. Stable overexpression of Drp1 via AAV under the control of the Drp1 promoter by intraspinal injection (Drp1 OE) attenuated abnormal gait and alleviated pain hypersensitivity of SNI mice. Mitochondrial ultrastructure analysis showed that the increased density of mitochondria induced by SNI was recovered by Drp1 OE which, however, did not change mitochondrial morphology and vacuole parameters within SDH. Contrary to Drp1 OE, down-regulation of Drp1 in the SDH by AAV-Drp1 shRNA (Drp1 RNAi) did not alter painful behavior induced by SNI. Ultrastructural analysis showed the treatment by combination of SNI and Drp1 RNAi (SNI + Drp1 RNAi) amplified the damages of mitochondria with the decreased distribution density, increased perimeter and area, as well as larger circularity tending to be more circular. Vacuole data showed SNI + Drp1 RNAi increased vacuole density, perimeter and area within the SDH mitochondria. Our results illustrate that mitochondria within the SDH are sensitive to NP, and targeted mitochondrial Drp1 overexpression attenuates pain hypersensitivity. Drp1 offers a novel therapeutic target for pain treatment.

## Highlights

• SNI induces pain hypersensitivity and increases Drp1 within SDH in mice.
• Targeted up-regulation of Drp1 in SDH attenuates SNI-pain hypersensitivity.
• Targeted up-regulation of Drp1 restores mitochondrial fragmentation and swelling.
• Targeted down-regulation of Drp1 does not alleviate pain with the deteriorated mitochondrial damages.
• Drp1 offers a novel therapeutic target for pain treatment.

## Graphical abstract

**Figure:**
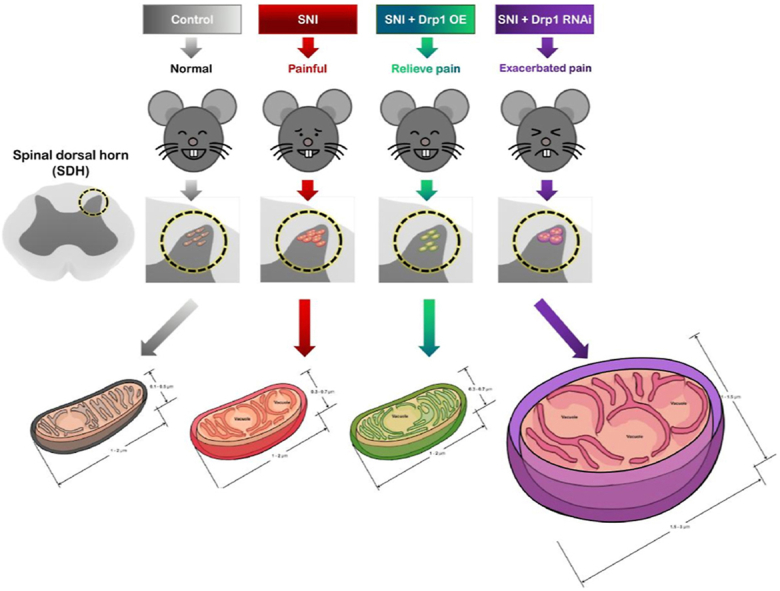

## Abbreviations

**Acronym Full name**
AAV: Adeno-associated virus
AD: Alzheimer's disease
ASO: Antisense oligodeoxynucleotide
CCI: Chronic constriction injury
CFA: Complete Freund's adjuvant
CPN: Common peroneal nerve
DRG: Dorsal root ganglion
Drp1: Dynamin-related protein 1
EPM: Elevated plus-maze
GABA: gamma-aminobutyric acid
IACUC: Institutional Animal Care and Use Committee
IpsiSDH: Ipsilateral spinal dorsal horn
IMM: Inner mitochondrial membrane
LF: Left front paw
LH: Left hind paw
Mfn1: Mitofusin 1
Mfn2: Mitofusin 2
Mito: Mitochondria
MitoQ: Mitoquinone
NP: Neuropathic pain
OE: Overexpression
OFT: Open field test
Opa1: Optic atrophy-1
OMM: Outer mitochondrial membrane
PBN: N-*tert*-Butyl-alpha-phenylnitrone
PD: Parkinson's disease
PTP: Permeability transition pore
PWT: Paw withdrawal threshold
qRT-PCR: Real time-quantitative PCR
RF: Right front paw
RH: Right hind paw
ROS: Reactive oxygen species
RNAi: RNA interference
SDH: Spinal dorsal horn
SN: Sural nerve
SNI: Spared nerve injury
SNL: Spinal nerve ligation
TEM: Transmission electron microscopy
TEMPOL: 4-hydroxy-2,2,6,6-tetramethylpiperidin-1-oxyl
TN: Tibial nerve
Vac: Vacuole
Vin: Vincristine

## 1 Introduction

Neuropathic pain (NP) is a kind of pain caused by peripheral or central nervous system lesions or injuries, and the patients with NP are often accompanied with a variety of symptoms such as paroxysmal pain, allodynia and hyperalgesia [1,2]. Due to the complex mechanism of NP, treatment of NP is still a challenge in clinical medicine [[3], [4], [5]]. SNI, ligation and axotomy of the tibial and common peroneal nerves but sural nerve intact, was reported to be a classic NP model [6,7]. The symptoms of allodynia and hyperalgesia in SNI mice were similar to the patients with NP. So we applied SNI model in the present study.

The spinal dorsal horn (SDH) receiving pain signal input from peripheral sensory neurons plays a pivotal position in the integration of pain signals and central pain sensitization [8,9]. Long-lasting changes in the processing of nociceptive information within the SDH contribute to pain-related pathological changes [9,10]. Our group have devoted to the study of the synaptic connectivity that underlies the noxious transmission and modulation within the SDH [11]. Here we focused on the plasticity changes of SDH in SNI model.

Mitochondria are crucial in the nervous system, especially in neurons [12,13]. They are the most sensitive device to detect the stability of neuron microenvironment. It is reported that mitochondria dysfunctions are closely related to various nervous system diseases via different mechanisms including oxidation respiration, mitochondrial dynamics, mitophagy, mitochondrial biogenesis and calcium homeostasis [14,15]. Importantly, neuronal mitochondrial function exceptionally depend on stable mitochondrial dynamics of fission and fusion, which shapes mitochondrial morphology and architecture [[15], [16], [17], [18]]. Excessive mitochondrial fragmentation or swollen mitochondria with expanded vacuole space and fewer cristae, or disruption of the mitochondrial network, has been indicated to be associated with neurological diseases [15,18,19]. Mitochondrial division is mediated by the conserved dynamin-related GTPase Drp1 which assembles onto the surface of mitochondria and constricts this tubular organelle [20]. Drp1 has been shown to be required for the development of nervous system and Drp1 deletion causes embryonic lethality in mice [21,22]. Moreover, mitochondrial division regulated by Drp1 is essential for the suppression of oxidative damage and therefore for the survival of postmitotic neurons both in vivo and in vitro [22,23]. However, it is not known how the mitochondrial component within SDH is responsive under the SNI pathological conditions and what effect of up-regulation or down-regulation of Drp1 is on pain behaviors.

Herein, we explored the effects of up- and down-regulation of Drp1 within the SDH on animal gait and pain behaviors. We also studied parameters associated with the mitochondrial ultrastructure in different groups. The present results suggest that Drp1 is the potential molecule attenuating pain in SNI model.

## 2 Results

### 2.1 SNI induced pain behavior and anxiety-like behavior

**Fig. 1:**
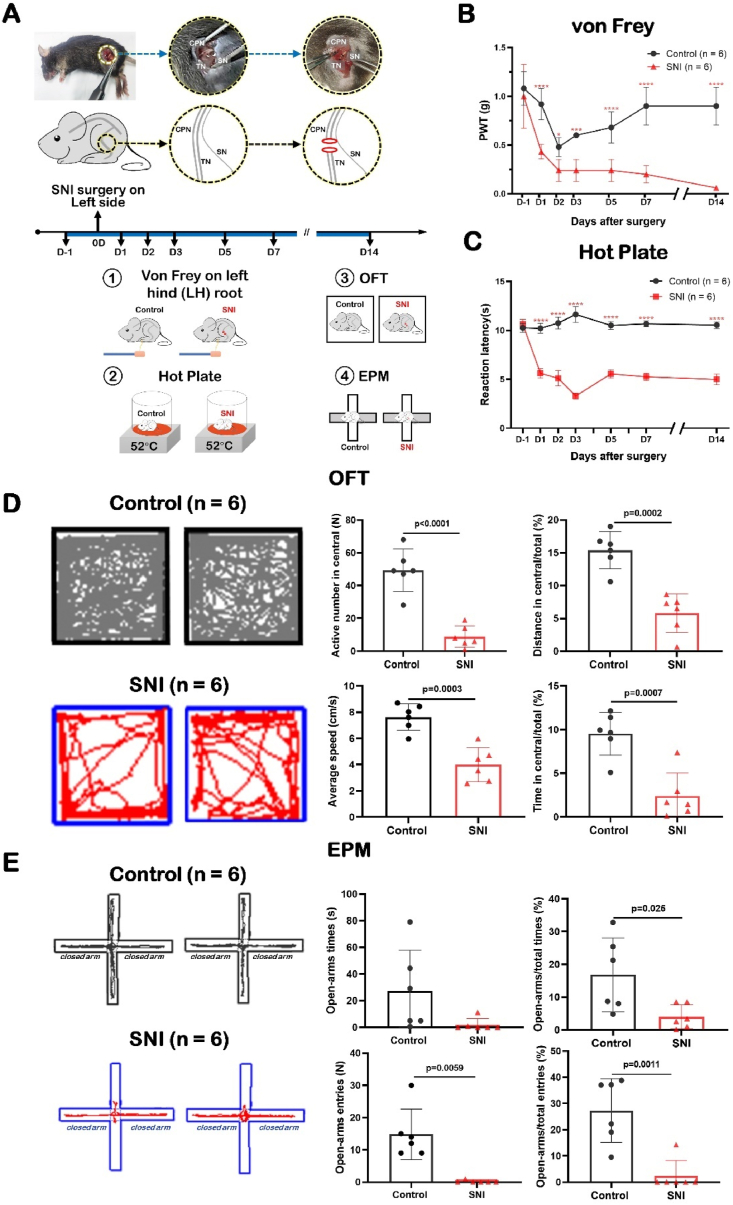
SNI induced pain behavior and anxiety-like behavior. Data are presented as mean ± S.D. See Supplemental Tables 1 and 2 for detailed information. *P < 0.05, **P < 0.01, ***P < 0.001, ****P < 0.0001. SNI: spared nerve injury; PWT: paw withdrawal threshold; OFT: open field test; EPM: elevated plus-maze. (A) Experimental timeline and schematic diagram of SNI modeling and behavioral testing in C57BL/6 mice. (B, C) The PWT of von Frey and reaction latencies to hot plate test showing SNI exacerbated ipsilateral mechanical sensitivity and thermal hyperalgesia in C57BL/6 mice (n = 6 for each group). Kruskal-Wallis *H* test with Nemenyi multiple comparisons test. (D) Traveling trajectory in the OFT and quantitative summary showing SNI mice spent less time and shorter distances in the middle of the open field (n = 6). Two-tailed unpaired separate variance estimation *t*-test. (E) Traveling trajectory in the EPM and quantitative summary showing SNI mice traveled less time and distance in open arm (n = 6). Two-tailed unpaired separate variance estimation *t*-test.

Pathophysiology of NP is sophisticated, and a desire to understand the underlying pathophysiological mechanisms entails the construction of preclinical models that mimic, as closely as possible, human clinical NP symptoms [24]. Here, we concentrated on the Spared Nerve Injury (SNI) model that was developed by Decostered and Woolf [6], which is highly characterized on both the sensory (injurious) and affective (emotional) dimensions, and induces long-term painful behavior and dysfunctions. With von Frey test, the golden standard for algesimetry test, changes in mechanical sensitivity of the paw (paw withdrawal threshold, PWT) have been assessed by our lab [25]. Here we firstly confirmed SNI induced adult mice to show persistent hypersensitivity and allodynia in the lateral paw skin area innervated by the sural nerve (Fig. 1A). PWT scores decreased markedly after SNI (D1, control: 0.93 ± 0.16 g; SNI: 0.36 ± 0.10 g; P < 0.0001) and remained significantly weaker than those of control mice for 2 weeks (D14, control: 0.870 ± 0.21 g; SNI: 0.06 ± 0.02 g; P < 0.0001) (Fig. 1B). In addition, the reaction latency also showed the similar decreased trend (D1, control: 10.21 ± 0.52 s; SNI: 5.62 ± 0.49 s; P < 0.0001. D14, control: 10.54 ± 0.34 s; SNI: 4.99 ± 0.23 s; P < 0.0001), suggesting that SNI caused a significant reduction in heat pain threshold (Fig. 1C).

In addition, the mice with NP also showed anxiety-like behavior (Fig. 1D and E), which was consistent with previous report [26]. Rodents with anxiety are afraid to explore surroundings and tend to stay in a safer place, which is the periphery of the open field and the enclosed arms of EPM [27,28]. By using open field test (OFT), our results showed that the number of activities in the central region for mice subjected to SNI (control: 49.33 ± 12.97; SNI: 8.83 ± 6.55; P < 0.0001), accompanied by the percentage of distance (control: 15.39 ± 2.83%; SNI: 5.79 ± 2.93%; P = 0.0002) and time (control: 9.54 ± 2.46%; SNI: 2.38 ± 2.63%; P = 0.0007) in central area of OFT were significantly reduced, which supported an increase in anxiety-like behavior (Fig. 1D). And the average speed in OFT was decreased remarkably in SNI mice, compared with the control group. By using elevated plus-maze (EPM), the SNI mice also showed anxiety-like behavior (Fig. 1E). The results showed that the percentage of time to open-arm significantly decreased (control: 16.84 ± 11.28%; SNI: 4.05 ± 3.67%; P = 0.025), the number of open-arm entries (control: 14.83 ± 7.83; SNI: 0.17 ± 0.41; P = 0.0059) and the percentage of open-arm entries (control: 27.29 ± 12.11%; SNI: 2.38 ± 5.83%; P = 0.0011) of EPM were significantly lower in the SNI group compared to the control group (Fig. 1E).

These results showed SNI induced pain behavior and anxiety-like behavior in mice.

### 2.2 SNI induced morphological changes and ultrastructure damage of mitochondria in SDH

**Fig. 2:**
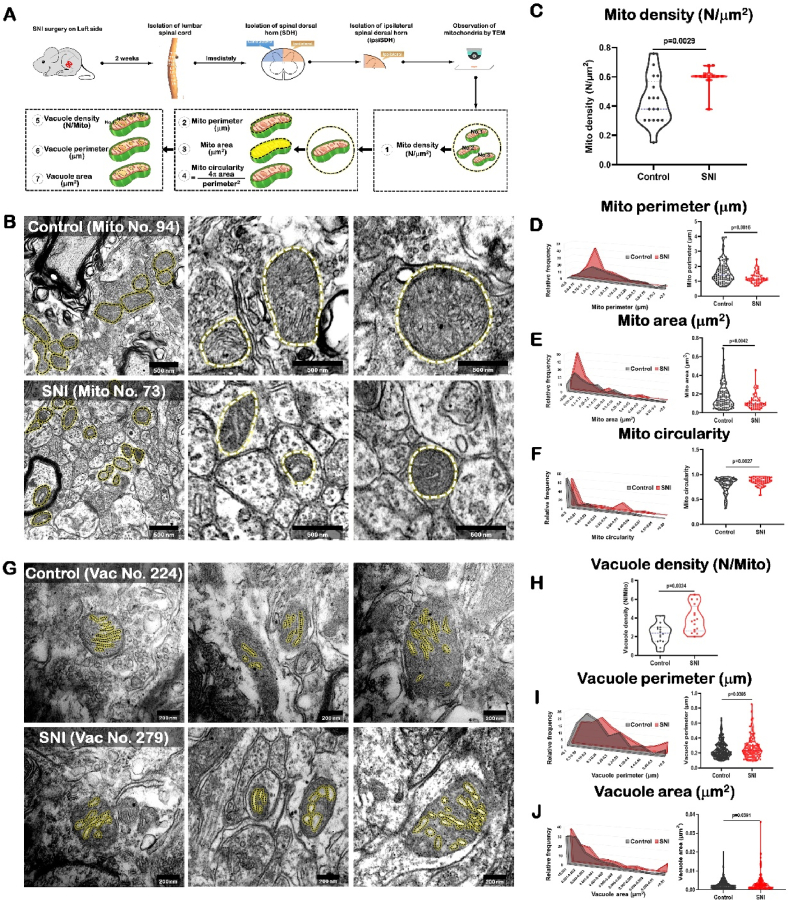
SNI induced morphological changes and ultrastructure damage of mitochondria in SDH. Data are presented as mean ± S.D. SNI: spared nerve injury; Mito: mitochondria; Vac: vacuole. (A) Schematic diagram for tissue sampling and electron microscope data collection of spinal dorsal horn of SNI mice. (B) Representative electron microscopic images of mitochondria for spinal dorsal horn neurons in control (top, n = 94) and SNI (bottom, n = 73) mice. Bright dotted lines indicate mitochondria. Scale bar, 500 nm (C) Quantitative analysis showed that the mitochondrial density of SNI mice (n = 17) was lower than that of control mice (n = 12). Two-tailed unpaired separate variance estimation *t*-test. (D) Diagram of distribution pattern of mitochondrial perimeter (left) and quantitative analysis showed that the mitochondrial perimeter of SNI mice (n = 73) was shorter than that of control mice (n = 94) (right). Two-tailed unpaired separate variance estimation *t*-test. (E) Diagram of distribution pattern of mitochondrial area (left) and quantitative analysis showed that the mitochondrial area of SNI mice (n = 73) was smaller than that of control mice (n = 94) (right). Two-tailed unpaired separate variance estimation *t*-test. (F) Diagram of distribution pattern of mitochondrial circularity (left) and quantitative analysis showed that the circularity of mitochondrial of SNI mice (n = 73) was higher than that of control mice (n = 94) (right). Two-tailed unpaired separate variance estimation *t*-test. (G) Representative electron microscopic images of mitochondria vacuoles for spinal dorsal horn neurons in control (top, n = 224) and SNI (bottom, n = 279) mice. Bright dotted lines indicate vacuoles in the mitochondria. Scale bar, 200 nm (H) Quantitative analysis showed that the density of mitochondria vacuoles of SNI mice (n = 16) was higher than that of control mice (n = 16). Two-tailed unpaired separate variance estimation *t*-test. (I) Diagram of distribution pattern of vacuoles perimeter in mitochondria (left) and quantitative analysis showed that the vacuoles perimeter in mitochondria of SNI mice (n = 224) was longer than that of control mice (n = 279) (right). Two-tailed unpaired separate variance estimation *t*-test. (J) Diagram of distribution pattern of vacuoles area in mitochondria (left) and quantitative analysis showed that the vacuoles area in mitochondria of SNI mice (n = 224) was larger than that of control mice (n = 279) (right). Two-tailed unpaired separate variance estimation *t*-test.

Mitochondria are kept in vigilant thought to be a multi-layered quality control system that shields them from various stresses and ensures the maintenance of healthy mitochondria [29,30]. There are no studies that evaluate the changes of mitochondrial dynamics following the pain. Our lab has investigated the mitochondrial distribution and subcellular morphology in SNI mice by using the mitochondrial marker, Mitotracker Red® CM-H2XRox (Mito-Red) [31]. In order to exactly visualize the architectural changes of SDH mitochondria following SNI, the transmission electron microscopy (TEM) was used to measure seven parameters which is the combined method from previous reports [19,32]. In detail, the seven important mitochondrial parameters were used, including: a. mitochondria density (mito density; N/μm^2^); b. mitochondria perimeter (mito perimeter; μm); c. mitochondria area (mito area; μm^2^); d. mitochondrial circularity; e. vacuole density (N/mito); f. vacuole perimeter (μm); g. vacuole area (μm^2^) (Fig. 2A).

**Fig. 3:**
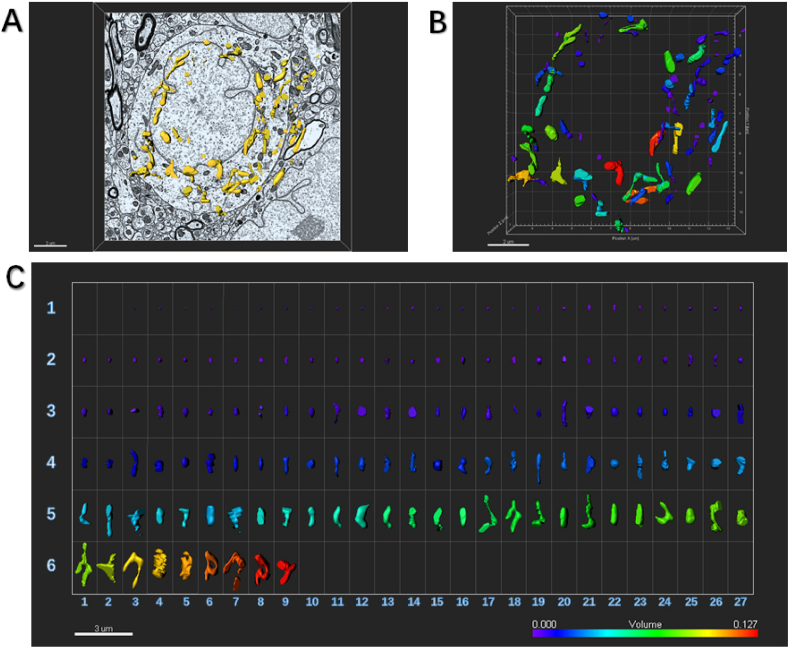
An example to show how an image of electron microscopy block captured from the SDH in the control mouse is processed for serial 3-dimensional reconstructions. SDH: spinal dorsal horn. (A) Imaris software was used to reconstruct SDH in the volume of 16 × 15.5 × 1.75 μm from 35 electron microscope images of continuous sections. Scale bar, 2 μm (B) The Vantage module of Imaris was used to establish three-dimensional reconstruction of mitochondria in the SDH of control mice, and the plot type was presented in XYZ color format. Scale bar, 2 μm (C) Three-dimensional mitochondria were presented in Gallery format and arranged according to volume. Scale color was set according to the volume from purple indicating the minimum volume of mitochondria of 0.000 μm^3^ to the red marking the maximum volume of mitochondria of 0.127 μm^3^. In the Gallery format in the control group, there were 144 mitochondria captured from one neuronal soma in SDH. We can see 106 mitochondria had small volume ranged from 0.001 μm^3^ to 0.050 μm^3^, 32 mitochondria had moderate volume ranged from 0.051 μm^3^ to 0.088 μm^3^, and 8 eight mitochondria had large volume ranged from 0.089 μm^3^ to 0.127 μm^3^. Scale bar, 3 μm. (For interpretation of the references to color in this figure legend, the reader is referred to the Web version of this article.)

**Fig. 4:**
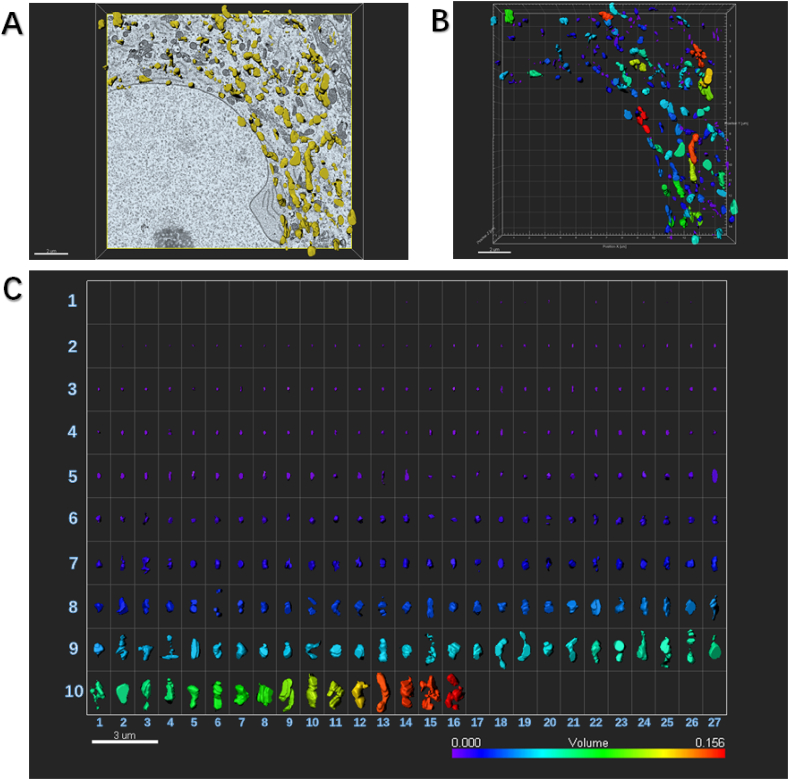
An example to show how an image of electron microscopy block captured from the ipsilateral SDH in the SNI mouse is processed for serial 3-dimensional reconstructions. SNI: spared nerve injury; SDH: spinal dorsal horn. (A) Imaris software was used to reconstruct SDH in the volume of 16 × 15.5 × 1.75 μm from 35 electron microscope images of continuous sections. Scale bar, 2 μm (B) The Vantage module of Imaris was used to establish three-dimensional reconstruction of mitochondria in the SDH of SNI mice, and the plot type was presented in XYZ color format. Scale bar, 2 μm (C) Three-dimensional mitochondria were presented in Gallery format and arranged according to volume. Scale color was set according to the volume. In the SNI group, there were more number of mitochondria of 259 captured from one neuronal soma in the ipsilateral SDH. It should be noted that these mitochondria tended to be much smaller. Of 259 captured mitochondria, 216 mitochondria (83%) had small volume ranged from 0.001 μm^3^ to 0.062 μm^3^, 35 mitochondria (14%) had moderate volume ranged from 0.063 μm^3^ to 0.010 μm^3^, and 8 mitochondria (3%) had large volume from 0.011 μm^3^ to 0.156 μm^3^. Scale bar, 3 μm. (For interpretation of the references to color in this figure legend, the reader is referred to the Web version of this article.)

The results showed that compared with the control mice, the density of mitochondria in neurons increased (control: 0.43 ± 0.16/μm^2^; SNI: 0.60 ± 0.08/μm^2^; P = 0.0029), and the perimeter and area of mitochondria of mice after SNI became shorter and smaller (Perimeter, control: 1.61 ± 0.71 μm; SNI: 1.25 ± 0.37 μm; P = 0.0016. Area, control: 0.18 ± 0.13 μm^2^; SNI: 0.12 ± 0.08 μm^2^; P = 0.0042), suggesting that mitochondrial division increased and the number increased after SNI (Fig. 2B–E; Fig. 3; Fig. 4; Video 1; Video 2).

Supplementary video related to this article can be found at https://doi.org/10.1016/j.redox.2021.102216

The following are the supplementary data related to this article:

Multimedia component 1 Imaris software was used to reconstruct mitochondria located in one neuronal cell within the spinal dorsal horn (SDH) of normal control mouse. Electron microscope sections of the tissue were selected and 35 continuous electron microscope sections with a volume of 16 × 15.5 × 1.75 μm^3^ were reconstructed using Imaris software. About 144 mitochondria (yellow) in neurons were labeled with the surface creation. Related to Figure 2B and Fig. 3. Multimedia component 1

Multimedia component 2 Imaris software was used to reconstruct mitochondria located in one neuronal cell within the SDH of SNI mouse. Electron microscope sections of the tissue were selected and 35 continuous electron microscope sections with a volume of 16 × 15.5 × 1.75 μm^3^ were reconstructed using Imaris software. About 259 mitochondria (yellow) in neurons were labeled with the surface creation. SNI: spared nerve injury; SDH: spinal dorsal horn. Related to Fig. 2 G and Fig. 4. Multimedia component 2

We also introduced the index of mitochondrial circularity, a measure that correlates well with mitotoxicity and neurotoxicity [32], into the present study. The results showed that the circularity of mitochondria increased significantly after SNI (control: 0.79 ± 0.15; SNI: 0.87 ± 0.07; P = 0.0027) (Fig. 2F), suggesting that the mitochondrial health status was impaired.

In addition, the present study was to analyze the mitochondrial vacuoles which reflect the damage state of the inner mitochondrial membrane into many separate vesicular matrix compartments [19]. The statistical results of mitochondrial vacuoles showed that the perimeter and area of mitochondrial vacuoles increased (Perimeter, control: 244.0 ± 108.5 nm; SNI: 269.6 ± 141.3 nm; P = 0.0386. Area, control: 2893.1 ± 2555.2 nm^2^; SNI: 3676.4 ± 4855.2 nm^2^; P = 0.0361) in mice with NP, and the density of mitochondria vacuoles was higher (control: 2.39 ± 1.10/mito; SNI: 3.90 ± 1.45/mito; P = 0.0024) (Fig. 2G–J). The results showed that SNI induced damage of the inner membrane structures within the SDH mitochondria.

Here we specially established three-dimensional reconstruction of mitochondria in the ipsilateral SDH from control (Fig. 3) and SNI (Fig. 4) mice, and analyzed the plot types captured in the volume of 16 × 15.5 × 1.75 μm from 35 electron microscope images of continuous sections, respectively. In the Gallery format in the control group (Fig. 3), there were 144 mitochondria captured from one neuronal soma in SDH. We could see 106 mitochondria (74%) had small volume ranged from 0.001 μm^3^ to 0.050 μm^3^, 32 mitochondria (22%) had moderate volume ranged from 0.051 μm^3^ to 0.088 μm^3^, and 8 mitochondria (4%) had large volume ranged from 0.089 μm^3^ to 0.127 μm^3^. However, in the SNI group a larger number of mitochondria were captured, 259 from one neuronal soma in the ipsilateral SDH (Fig. 4). It should be noted that these mitochondria tended to be much smaller. Of 259 captured mitochondria, 83% mitochondria (216) had small volume ranged from 0.001 μm^3^ to 0.062 μm^3^, 14% mitochondria (35) had moderate volume ranged from 0.063 μm^3^ to 0.010 μm^3^, and only 3% mitochondria (8) had large volume from 0.011 μm^3^ to 0.156 μm^3^.

These results suggested the mitochondria within the SDH were sensitive to NP stress and tended to have the increased number and the decreased volume.

### 2.3 SNI led to the elevation of the mitochondrial fission factor Drp1 expression in SDH, but did not change the expression of two mitochondrial fusion factors of Mfn1 and Mfn2

**Fig. 5:**
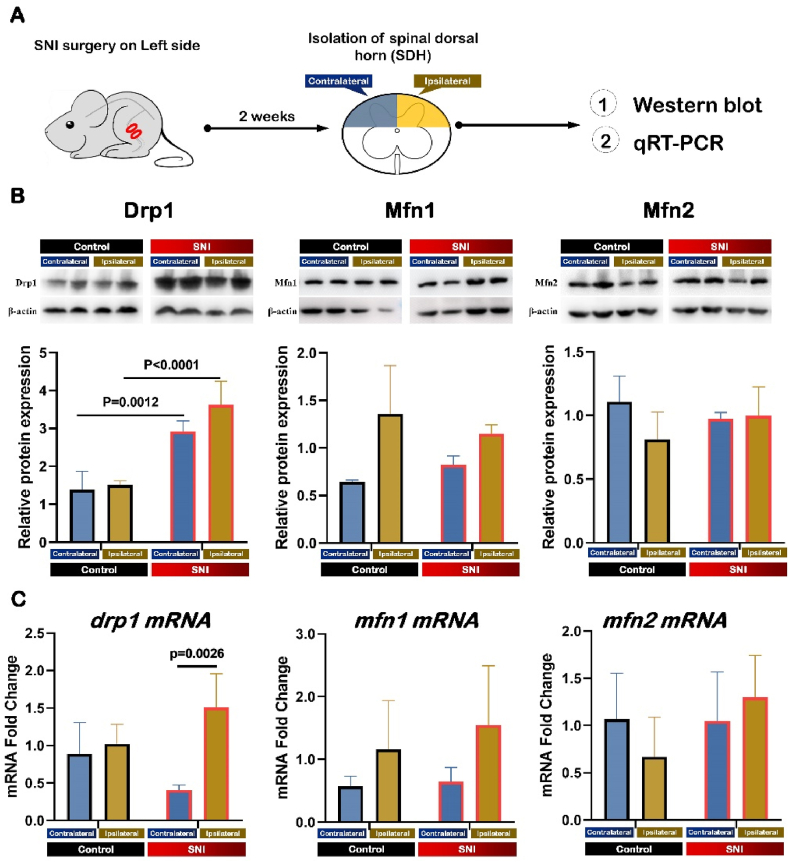
SNI led to the elevated expression of mitochondrial fission factor of Drp1 in SDH, but did not change the expression of mitochondrial fusion factors of Mfn1 and Mfn2. Data are presented as mean ± S.D. SNI: spared nerve injury; SDH: spinal dorsal horn. (A) Schematic diagram of tissue sampling from spinal dorsal horn of SNI mice. (B) Typical examples and quantitative summary levels of Drp1 (left), Mfn1 (middle) and Mfn2 (right) in the SDH tissue of the control (black border) and SNI (red border) mice using Western blot analysis. Data were normalized to the housekeeping protein β-actin (n = 4). Kruskal-Wallis *H* test with Nemenyi multiple comparisons test. (C) Quantitative summary levels of Drp1 (left), Mfn1 (middle) and Mfn2 (right) in the SDH tissue of the control and SNI mice using qRT-PCR. Kruskal-Wallis *H* test with Nemenyi multiple comparisons test. Blue columns indicate the ipsilateral side and yellow columns indicate the contralateral side. (For interpretation of the references to color in this figure legend, the reader is referred to the Web version of this article.)

Our previous study has shown that Drp1 is highly expressed at the spinal cord level [33]. And many studies have shown different NP contributed to the increased expression of Drp1 [[34], [35], [36], [37], [38], [39], [40], [41], [42]]. Here we observed the changes of Drp1 in the SDH of the mice during NP by Western blot and qRT-PCR and also confirmed the increased Drp1 expression (Fig. 5A).

The Western blot results showed that Drp1 protein was significantly up-regulated in ipsilateral SDH after SNI compared with that in control mice (control: 1.51 ± 0.11; SNI ipsilateral side: 3.62 ± 0.63; P < 0.0001) (Fig. 5B). Although Drp1 protein seemed higher on the ipsilateral side (3.62 ± 0.63) than that on the contralateral side (2.92 ± 0.28), there was no significant difference (P = 0.1428) (Fig. 5B).

The qRT-PCR results showed that Drp1 mRNAs were significantly up-regulated in ipsilateral SDH after SNI compared with those in control mice (control: 0.88 ± 0.42; SNI ipsilateral side: 1.51 ± 0.45; P < 0.0026) (Fig. 5C). Drp1 mRNAs were higher on the ipsilateral side than on the contralateral side (qRT-PCR: contralateral: 0.41 ± 0.07; ipsilateral: 1.51 ± 0.45; P = 0.0026) (Fig. 5C).

Mitochondrial fusion is the reverse regulation of fission, and the factors, Mitofusin 1/2 (Mfn1 and Mfn2), located on the outer mitochondrial membrane (OMM), are responsible for fusion process [43,44]. So we observed the changes of Mfn1 and Mfn2 in the SDH of mice during NP. The results showed that there was no significant difference in the expression of Mfn1 or Mfn2 in the spinal dorsal horn after SNI, either on the ipsilateral or the contralateral side (Fig. 5B and C).

These results suggested that elevated Drp1 may contribute to the number and morphological changes of mitochondria after SNI.

### 2.4 Targeted up-regulation of Drp1 within SDH alleviated pain symptoms in SNI mice, while targeted down-regulation of Drp1 did not have the analgesic effect

**Fig. 6:**
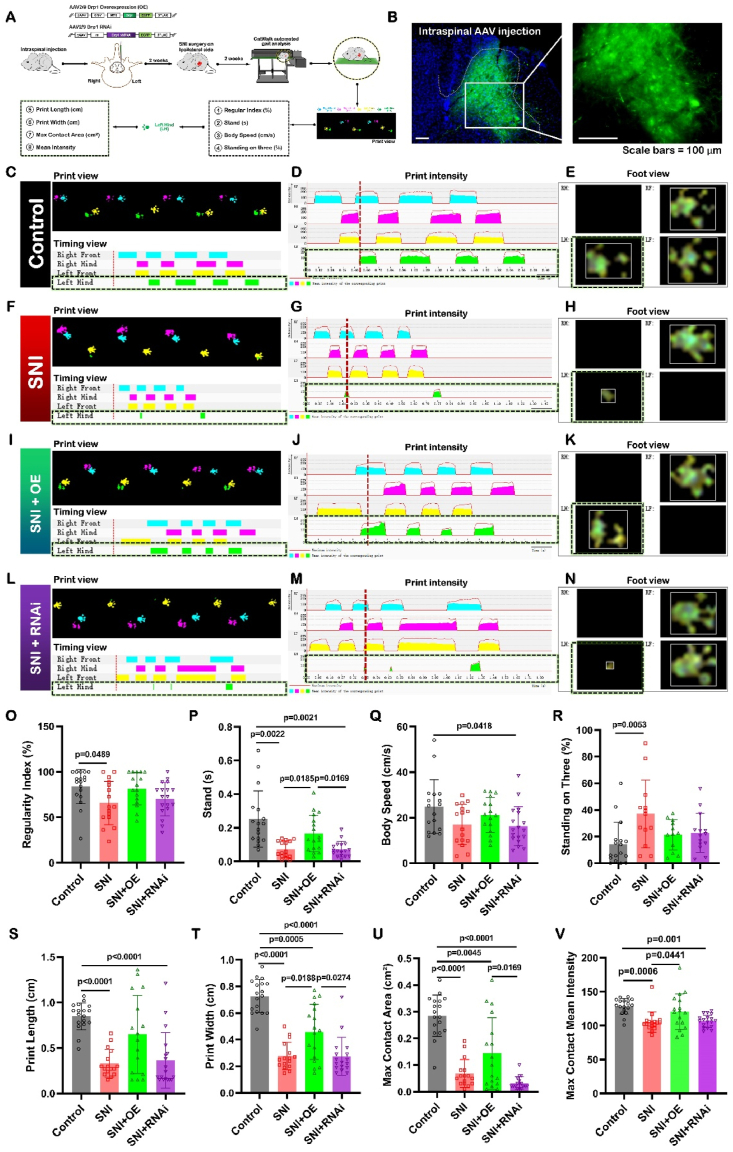
Targeted up-regulation of Drp1 within SDH alleviated pain symptoms in SNI mice; on the contrary, targeted down-regulation of Drp1 could not alleviate pain symptoms induced by SNI, analyzed by CatWalk gait method. Data were calculated as ipsilateral (left) hind paw. Data are expressed as mean ± SD. The number of detected animals is indicated inside of each column, by one-way ANOVA with post hoc Dunnett test. SNI: spared nerve injury; SDH: spinal dorsal horn; OE: overexpression; RNAi: RNA interference. (A) Schematic diagram showing intra-SDH virus injection in C57BL/6 mice and data collection from CatWalk gait test. (B) Confocal images of Drp1 overexpressed virus injected into the SDH of mice (n = 6). Scale bar, 100 μm. Representative CatWalk gait of print view and timing view (merged in C, F, I and L), area chart of print intensity (D, G, J and M), and foot view (E, H, K and N) for control group (C to E) (n = 6), SNI group (F to H) (n = 6), SNI + OE group (I to K) (n = 6), and SNI + RNAi group (L to N) (n = 6). We marked one single specific time point in the images (D, G, J and M) of area chart of print intensity and provided their associated foot views in images (E, H, K and N). It should be indicated that all black dotted lines in C–N marked the data from the ipsilateral (left) hind paw. (O–V) Statistical analysis of the eight parameters based on the CatWalk gait data. (O) Regularity index (%), a measure of inter-limb coordination. (P) Stand (s), the duration of a paw touching the glass plate. (Q) Body speed (cm/s), calculated by counting the distance of the mouse's body (paws) from the initial contact with the glass plate to the next contact divided by the time required to move this distance. (R) Standing on three (%), the percentage of time spent walking with three paws. (S) Print length (cm), the length of the paw print (horizontal direction). (T) Print width (cm), the width of the complete paw print (vertical direction). (U) Max contact area (cm^2^), the maximum print area during paw contact. (V) Max contact mean intensity, the mean intensity at maximum paw contact. We could find SNI induced significant decreases of the six parameters of regularity index (O), stand (P), print length (S), print width (T), max contact area (U), and max contact mean intensity (V); as well as induced significant increase of one parameter of standing on three (R). Unexpectedly, the targeted up-regulation of Drp1 by overexpression (OE) virus within SDH significantly alleviated decreased values of stand (P), print width (T), and max contact mean intensity (V) induced by SNI. On the contrary to the analgesia effect of Drp1 OE treatment, the targeted down-regulation of Drp1 by RNA interference (RNAi) virus within SDH could not make any improvement in seven parameters altered by SNI including regularity index (O), stand (P), print length (S), print width (T), max contact area (U), and max contact mean intensity (V), as well as standing on three (R). Behavioral tests were performed 2 weeks after the SNI surgery and 4 weeks after AAV delivery in Drp1 OE or RNAi group.

To verify the contribution of Drp1 to SNI-induced mechanical pain sensitization, two complementary methods were used to modulate the function of Drp1, as follows (Fig. 6A): (1) up-regulation of Drp1 repression with intraspinal injection of Drp1 overexpression (OE) AAV into the ipsilateral SDH 2 weeks prior to SNI surgery; and (2) down-regulation of Drp1 repression with intraspinal injection of Drp1 RNA interference (RNAi) AAV into the ipsilateral SDH 2 weeks prior to SNI surgery. Another 2 weeks after SNI, the spinal cord was isolated and studied by confocal microscopy observation, Western blot and qRT-PCR (Fig. 6A).

#### 2.4.1 Verification of up-regulation and down-regulation of Drp1 targeted to SDH in mice

The confocal imaging (Fig. 6B) confirmed the Drp1 OE AAV indeed fused into the ipsilateral SDH of SNI mouse. The RNAi AAV was also confirmed to be fused into the target of the ipsilateral SDH of SNI mouse by the confocal observation **(data not shown)**.

**Fig. 7:**
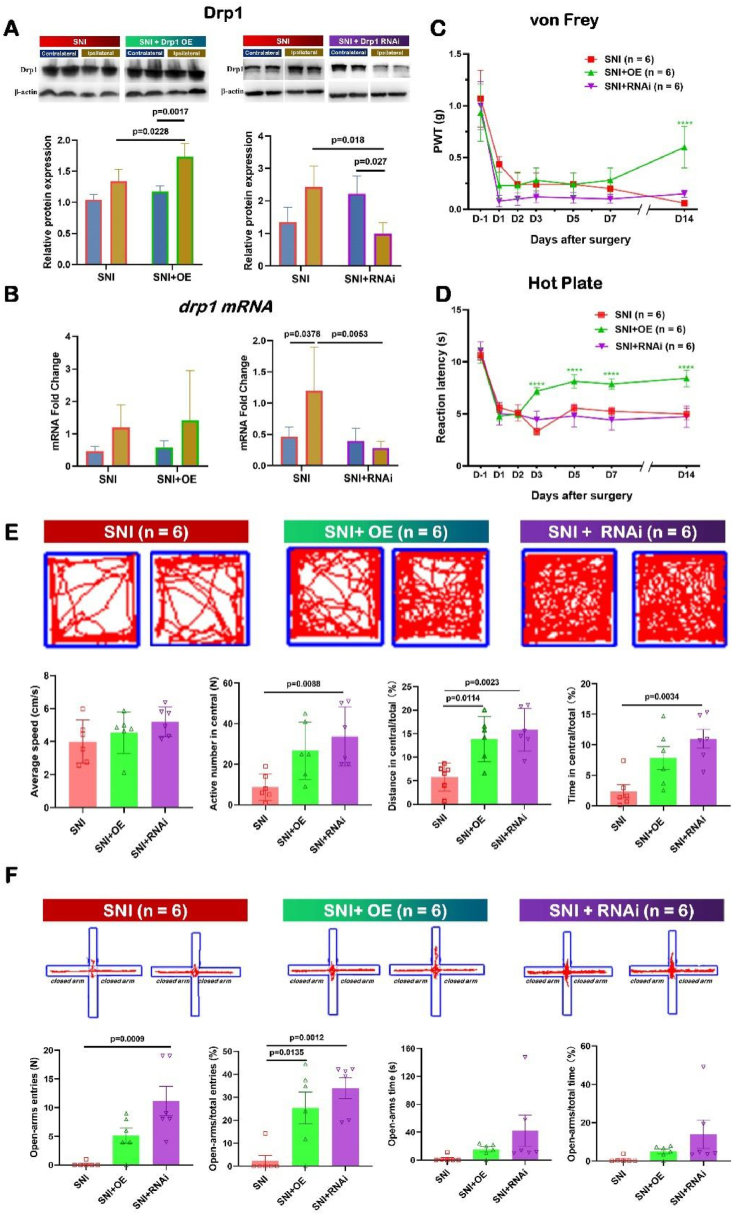
SNI-induced anxiety behavior could be partially alleviated with both Drp1 OE and Drp1 RNAi treatment by open field test (OFT) and elevated plus maze (EPM) methods. Data are presented as mean ± S.D. *P < 0.05, **P < 0.01, ****P < 0.0001. SNI: spared nerve injury; SDH: spinal dorsal horn; PWT: paw withdrawal threshold; OFT: open field test; EPM: elevated plus-maze. (A) Western blot analysis showed the expression of Drp1 protein in SNI mice after injection of overexpressed (left) and interfered (right) AAV into the SDH. Data were normalized to the housekeeping protein β-actin (n = 4). Kruskal-Wallis *H* test with Nemenyi multiple comparisons test. (B) Quantitative summary levels of Drp1 mRNA in SNI mice after injection of overexpressed (left) and interfered (right) AAV into the SDH using qRT-PCR. (C, D) The PWT tested by von Frey and reaction latencies to hot plate test showing overexpression of Drp1 alleviated ipsilateral pain-induced mechanical sensitivity and thermal hyperalgesia in SNI mice (n = 6 for each group). Kruskal-Wallis *H* test with Nemenyi multiple comparisons test. (E, F) Representative trajectory and quantitative summary of average speed, active number and time in center area in OFT (E), as well as entries and time in open arm in EPM (F) from SNI and AAV-treated mice (n = 6). Two-tailed unpaired separate variance estimation *t*-test.

In addition, Western blot results (Fig. 7A) verified the increased Drp1 expression at protein level induced by targeted Drp1 OE treatment, and the decreased Drp1 expression at protein level and mRNA level induced by targeted Drp1 RNAi treatment (OE, contralateral: 1.17 ± 0.09; ipsilateral: 1.73 ± 0.21; P = 0.0017. RNAi, contralateral: 2.22 ± 0.55; ipsilateral: 0.99 ± 0.34; P = 0.0027) (Fig. 7A).

Moreover, qRT-PCR results (Fig. 7B) verified the decreased Drp1 expression at mRNA level induced by targeted Drp1 RNAi treatment, when compared with the expression level of Drp1 mRNA in SNI group (SNI: 1.20 ± 0.70; SNI + RNAi: 0.28 ± 0.11; P = 0.0053) (Fig. 7B). The qRT-PCR results (Fig. 7B) showed the Drp1 expression at mRNA level increased from 0.58 ± 0.21 in SNI group to 1.41 ± 1.53 in SNI + OE group, but there was no statistical difference (Fig. 7B).

#### 2.4.2 Targeted up-regulation of Drp1 within SDH alleviated pain symptoms induced by SNI, analyzed by CatWalk gait method

Then we studied the effects of targeted up-regulation or down-regulation of Drp1 within SDH on pain symptoms in SNI mice. We have noticed that Ferrari LF et al. (2011) [41] have first reported that intrathecal administration of antisense oligodeoxynucleotide (ASO) against Drp1 markedly attenuated neuropathic mechanical hyperalgesia caused by anti-HIV/AIDS or anticancer chemotherapy in rats. In addition, Kanda H et al. (2015) [42] have reported that intrathecal administration of Drp1 ASO decreased mechanical allodynia in HIV NP in mice. Dai CQ et al. (2020) [45] have reviewed that block of Drp1 could be beneficial for pain animals.

In order to get the meticulous results about the item of the effect of drp1 on NP, we specifically introduced the CatWalk gait analysis which could provide exquisite and reliable observations for evaluating spontaneous hyperalgesia in pain model rodents [46,47]. CatwalkXT 10.6 automated gait analysis was used here.

We first performed Catwalk gait analysis of control and SNI group. Compared with the foot print data in control group (Fig. 6C–E), SNI pain-bearing mice showed remarkable changes (Fig. 6F–H). All seven parameters had significant differences between control group and SNI group (Fig. 6O–V), including: (1) regularity index (%) (Fig. 6O), a measure of inter-limb coordination; (2) stand (s) (Fig. 6P), the duration of a paw touching the glass plate; (3) standing on three (%) (Fig. 6R), the percentage of time spent walking with three paws; (4) print length (cm) (Fig. 6S), the length of the paw print (horizontal direction); (5) print width (cm) (Fig. 6T), the width of the complete paw print (vertical direction); (6) max contact area (cm^2^) (Fig. 6U), the maximum print area during paw contact; and (7) max contact mean intensity (Fig. 6V), the mean intensity at maximum paw contact; only except body speed (cm/s) (Fig. 6Q), which was calculated by counting the distance of the mouse's body (paws) from the initial contact with the glass plate to the next contact divided by the time required to move this distance. In detail, compared with the control group, SNI mice showed the significant increase in standing on three (control: 14.40 ± 16.39%; SNI: 37.13 ± 25.39%; P = 0.0053) (Fig. 6R), as well as the significant decrease in regularity index (control: 83.88 ± 19.02%; SNI: 65.86 ± 23.79%; P = 0.0489) (Fig. 6O), stand (control: 0.25 ± 0.17 s; SNI: 0.07 ± 0.05 s; P = 0.0022) (Fig. 6P), print length (control: 0.85 ± 0.15 cm; SNI: 0.33 ± 0.15 cm; P < 0.0001) (Fig. 6S), print width (control: 0.73 ± 0.12 cm; SNI: 0.28 ± 0.10 cm; P < 0.0001) (Fig. 6T), max contact area of the left posterior paw (ipsilateral) (control: 0.29 ± 0.08 cm^2^; SNI: 0.06 ± 0.04 cm^2^; P < 0.0001) (Fig. 6U), and max contact mean intensity (control: 128.6 ± 11.3; SNI: 105.4 ± 15.0; P = 0.0006) (Fig. 6V). So SNI pain-bearing mice showed remarkable painful behavioral changes analyzed by Catwalk method (Fig. 6F–H).

Secondly, we analyzed the effect of up-regulation of Drp1 by OE AAV on SNI-induced gait due to hyperalgesia situation. Compared that in SNI group, up-regulation of Drp1 could significantly improve the gait on three items: prolonged duration of stand (SNI: 0.07 ± 0.05 s; SNI + OE: 0.17 ± 0.11 s; P = 0.0185) (Fig. 6P), increased ipsilateral width (SNI: 0.28 ± 0.10 cm; SNI + OE: 0.46 ± 0.21 cm; P = 0.0188) (Fig. 6T), and increased max contact mean intensity (SNI: 105.4 ± 15.0; SNI + OE: 120.7 ± 21.2; P = 0.0441) (Fig. 6V). Drp1 OE treatment recovered the value of regularity index from 65.86 ± 23.79% in SNI condition to 81.58 ± 17.75% (Fig. 6O) which was close to the value at normal state, as well as recovered the value of standing on three from 37.13 ± 25.39% in SNI condition to 17.12 ± 13.47% (Fig. 6R) which was close to the value at normal state; there was no significant difference between SNI and SNI + OE. Similarly, the value of print length increased (SNI: 0.33 ± 0.15 cm; SNI + OE: 0.65 ± 0.43 cm) (Fig. 6S), and the value of ipsilateral max contact area also increased (SNI: 0.06 ± 0.04 cm^2^; SNI + OE: 0.11 ± 0.11 cm^2^ (Fig. 6U); there was no significant difference between SNI and SNI + OE. These results indicated the effects of targeted up-regulation of Drp1 within SDH could be beneficial for SNI pain symptoms in mice. Our present results were unexpectedly opposite with previous three reports mentioned in the last paragraph.

#### 2.4.3 Targeted down-regulation of Drp1 could not alleviate pain symptoms induced by SNI, analyzed by CatWalk gait method

In order to draw a clear conclusion about the effect of regulation of Drp1 on pain, we further packaged RNA interference (RNAi) rAAV against Drp1 and analyzed the effect of down-regulation of Drp1 by RNAi AAV on SNI-induced abnormal gait.

Thirdly, gait analysis data showed that down-regulation of Drp1 by RNAi could not make any improvement in seven parameters altered by SNI, including: regularity index (Fig. 6O), stand (Fig. 6P), standing on three (Fig. 6R), print length (Fig. 6S), print width (Fig. 6T), max contact area (Fig. 6U), and max contact mean intensity (Fig. 6V). The values of the seven parameters of the mice in SNI + RNAi group were: 70.07 ± 18.43% of regularity index (Fig. 6O), 0.09 ± 0.07 s of stand (Fig. 6P), 21.31 ± 15.26% of standing on three (Fig. 6R), 0.37 ± 0.30 cm of print length (Fig. 6S), 0.29 ± 0.14 cm of print width (Fig. 6T), 0.04 ± 0.05 cm^2^ of max contact area (Fig. 6U), and 108.28 ± 11.78 of max contact mean intensity (Fig. 6V). Based on these behavioral data, we could see the RNAi treated SNI mice avoided putting the ipsilateral paw on the plate, which was an indicator for being in the pain hypersensitive state.

#### 2.4.4 Targeted up-regulation of Drp1 within SDH alleviated pain symptoms induced by SNI, analyzed by von Frey and hot plate methods

To reconfirm the effect of Drp1 regulation on SNI pain, we carried out von Frey and hot plate in our further study, which are the classic and credible algesimetry of mechanical pain and heat pain, respectively [48]. Von Frey and hot plate were also conducted to detect whether silencing Drp1 aggravated the pain induced by SNI in spite of no difference shown in Catwalk gait analysis.

In the present study, the up-regulation of Drp1 remarkably increased the value of paw withdrawal threshold (PWT) reduced by SNI (Fig. 7C). In the first part of Results section (2.1), we have shown all SNI mice withdrew the ipsilateral hind paw once stimulated by the smallest von Frey filament equal to or below the threshold of 0.07 g, only within 5 s (Fig. 1B). However, compared with those in SNI group, the values of PWT of the mice in SNI + OE group significantly increased (Fig. 7C; SNI: 0.06 ± 0.02 g; SNI + OE: 0.60 ± 0.22 g; P < 0.0001). In detail, all SNI mice with the targeted treatment of Drp1 OE within the SDH did not withdraw the ipsilateral hind paw at the stimulation level of the smallest von Frey at 0.07 g (Fig. 7C). Until evoked with the smallest von Frey filament at 0.4 g, 2 SNI mice with the targeted Drp1 OE treatment began to withdraw the ipsilateral hind paw within 5 s. Moreover, 3 SNI mice with the targeted Drp1 OE treatment began to withdraw the ipsilateral hind paw within 5 s when evoked with the smallest von Frey filament at 0.6 g. In addition, 1 SNI mouse with Drp1 OE treatment withdrew the hind paw within 5 s, even when evoked with the smallest von Frey filament at 1.0 g.

Consistently, the reaction latency tested by hot plate also contributed to illustrating the effect of pain relief of the treatment of Drp1 overexpression (Fig. 7D). In the first part of Results section (2.1), all SNI mice were observed to hop from the 52 °C plate surface and lick the ipsilateral hind paw with the reaction latency of approximately 5 s. However, compared with that in SNI group, the reaction latency of the mice in SNI + OE group significantly increased (Fig. 7D; SNI: 4.99 ± 0.23 s; SNI + OE: 8.42 ± 0.79 s; P < 0.0001). In detail, all SNI mice with the targeted Drp1 OE hopped from the hot plate surface and licked the ipsilateral hind paw with the reaction latency longer than 6 s (Fig. 7D). Even 5 of 6 SNI + OE mice hopped and began to lick with the reaction latency longer than 8 s.

These results showed the targeted up-regulation of Drp1 within SDH could alleviate pain symptoms in SNI mice, when analyzed by von Frey and hot plate methods.

#### 2.4.5 Targeted down-regulation of Drp1 could not alleviate pain symptoms induced by SNI, analyzed by von Frey and hot plate methods

Compared with that in SNI group, the values of PWT in SNI + RNAi group increased, yet with no statistical difference between SNI group and SNI + RNAi group (Fig. 7C; SNI: 0.06 ± 0.02 g; SNI + RNAi: 0.15 ± 0.04 g; P = 0.4862). Further in thermal hypersensitivity testing, compared with those in SNI group, the values of reaction latency in SNI + RNAi group did not have any increase (Fig. 7D; SNI: 4.99 ± 0.23 s; SNI + RNAi: 4.73 ± 1.03 s; P > 0.5887). Even 2 of 6 SNI + RNAi mice hopped and began to lick with the reaction latency shorter than 3.74 s.

These results showed the targeted down-regulation of Drp1 within SDH could not make any improvement in alleviating pain symptoms in SNI mice, when analyzed by von Frey and hot plate methods.

### 2.5 SNI-induced anxiety behavior could be partially alleviated by both Drp1 OE and Drp1 RNAi treatment

It is generally believed that there is an interaction between nociceptive and anxiety-like behavior in rodents, so relieving pain in NP animals can also reverse NP-induced anxiety-like behavior [[49], [50], [51]]. For the purpose of exploring the emotional changes of targeted regulation of Drp1, we compared the Drp1 OE and Drp1 RNAi respectively with the SNI group using OFT and EPM.

In our study, we found that the ratio of distance moved in the central and total area (distance in central/total, %), one of the four indicators of OFT, showed increase in the Drp1 OE group (Fig. 7E; SNI: 5.79 ± 2.94; SNI + OE: 13.88 ± 4.82; P = 0.0014). And the other 3 indicators showed no significant difference but a rising trend between SNI and Drp1 OE group, containing: average speed (cm/s) (Fig. 7E; SNI: 4.00 ± 1.30; SNI + OE: 4.54 ± 1.26; P = 0.7035), active number in central region (N) (Fig. 7E; SNI: 8.83 ± 6.55; SNI + OE: 26.67 ± 14.11; P = 0.0597) and the ratio of duration in central and total area (time in central/total, %) (Fig. 7E; SNI: 2.38 ± 2.63; SNI + OE: 7.85 ± 4.62; P = 0.0573).

As for EPM testing, up-regulation of Drp1 increased the percentage of open-arm entries (open-arm/total entries, %) of EPM ranging from 2.38 ± 5.83 to 25.42 ± 16.91 (P = 0.0135) (Fig. 7F). Similarly, other 3 indicators of EPM between SNI and Drp1 OE group showed no difference, including open-arms entries (N) (Fig. 7E; SNI: 0.17 ± 0.41; SNI + OE: 5.17 ± 3.25; P = 0.1211), open-arms time (s) (Fig. 7E; SNI: 2.01 ± 4.53; SNI + OE: 15.25 ± 8.95; P = 0.7605) and the percentage of open-arms time in total time (open-arms/total time, %) (Fig. 7E; SNI: 0.67 ± 1.51; SNI + OE: 5.08 ± 2.98; P = 0.7605).

It was foreseeable that SNI-induced anxiety behavior was partially alleviated in Drp1 OE mice.

For the Drp1 RNAi group, OFT testing first showed it led to significant increase in the active number in central region (Fig. 7E; SNI: 8.83 ± 6.55; SNI + RNAi: 33.67 ± 14.65; P = 0.0088), the percentage of distance in central (Fig. 7E; SNI: 5.79 ± 2.94; SNI + RNAi: 15.81 ± 4.53%; P = 0.0023) and the percentage of time in central area of OFT (Fig. 7E; SNI: 2.38 ± 2.63; SNI + RNAi: 10.96 ± 3.74; P = 0.0034). A second EPM testing showed Drp1 RNAi treatment significantly increased the number of open-arm entries in EPM (Fig. 7F; SNI: 0.17 ± 0.41; SNI + RNAi: 11.17 ± 6.31; P = 0.0009), and the percentage of open-arm entries of EPM (Fig. 7F; SNI: 2.38 ± 5.83; SNI + RNAi: 33.97 ± 11.25; P = 0.0012). And in Drp1 RNAi group, the average speed in OFT, open-arm times in EPM and the percentage of open-arm times of EPM showed no statistical difference (Fig. 7F), when compared with those in SNI group.

It was unforeseeable that SNI-induced anxiety behavior was partially alleviated by Drp1 RNAi treatment considering its unbeneficial effect on abnormal gait and hyperalgesia induced by SNI.

We thought these results were possible because previous reports have shown that SNI pain was independent of emotional alternation and pathway [52,53]. It was proved that the neural conduction involved in anxious behavior and the neuronal activation required for were separate. Here we proposed that the effect of Drp1 regulation on pain should mainly depend on the exact mitochondrial changes of both ultrastructure morphology and vacuole parameters. So we compared and analyzed the three-dimensional reconstruction of mitochondria in different groups in the next step.

### 2.6 Up-regulation of Drp1 reduced mitochondrial number, but did not change the external and internal appearance of the mitochondria induced by SNI; on the contrary, down-regulation of Drp1 led SDH mitochondria to be more swollen and with more broken cristae

**Fig. 8:**
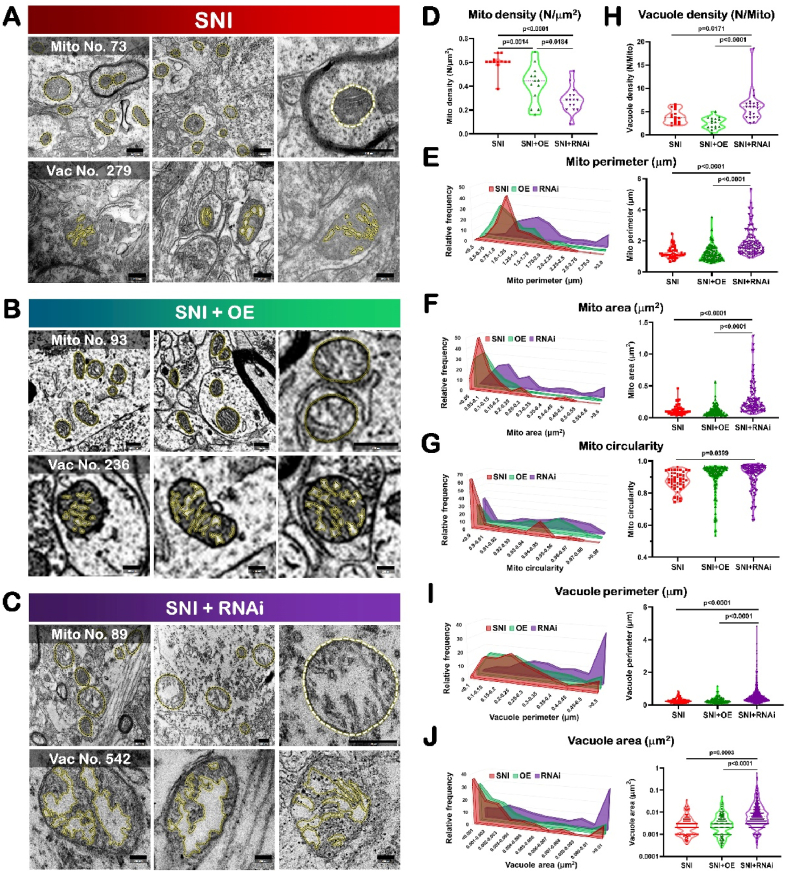
Up-regulation of Drp1 reduced mitochondrial number, but did not change the external and internal appearance of the mitochondria induced by SNI; on the contrary, down-regulation of Drp1 led SDH mitochondria to be more swollen and with more broken cristae. Data are presented as mean ± S.D. SNI: spared nerve injury; SDH: spinal dorsal horn; OE: overexpression; RNAi: RNA interference; Mito: mitochondria; Vac: vacuole. (A) Representative electron microscopic images of mitochondria (top, n = 73) and mitochondrial vacuoles (bottom, n = 279) for spinal dorsal horn neurons in SNI mice. Top, bright dotted lines indicate mitochondria. Scale bar, 500 nm. Bottom, bright dotted lines indicate vacuoles in the mitochondria. Scale bar, 200 nm. (B) Representative electron microscopic images of mitochondria (top, n = 93) and mitochondrial vacuoles (bottom, n = 236) for spinal dorsal horn neurons in SNI + Drp1 OE mice. Top, bright dotted lines indicate mitochondria. Scale bar, 500 nm. Bottom, bright dotted lines indicate vacuoles in the mitochondria. Scale bar, 200 nm. (C) Representative electron microscopic images of mitochondria (top, n = 89) and mitochondrial vacuoles (bottom, n = 542) for spinal dorsal horn neurons in SNI + Drp1 RNAi mice. Top, bright dotted lines indicate mitochondria. Scale bar, 500 nm. Bottom, bright dotted lines indicate vacuoles in the mitochondria. Scale bar, 200 nm.

It was well-known that Drp1 was involved in various neurological diseases by regulating the homeostasis of mitochondrial fission and fusion process [15,18,19]. So we further explored and analyzed the mitochondrial morphology and dynamics in detail considering the obvious behavioral changes after targeted regulation of Drp1 (Fig. 8).

We knew SNI induced more mitochondria densely distributed within the ipsilateral SDH (Fig. 2, Fig. 4, Fig. 8A; Video 2), and these mitochondria appeared with longer perimeter (Fig. 2, Fig. 4, Fig. 8A; Video 2), larger area (Fig. 2, Fig. 4, Fig. 8A; Video 2), and increased circularity (Fig. 2, Fig. 4, Fig. 8A; Video 2), which implied these mitochondria turned to be in sphere shape. The statistical results of mitochondrial vacuoles showed that SNI induced more distributed vacuole located in mitochondria within the ipsilateral SDH (Fig. 2, Fig. 4, Fig. 8A; Video 2); and these vacuole appeared with the longer perimeter (Fig. 2, Fig. 4, Fig. 8A; Video 2), and larger area (Fig. 2, Fig. 4, Fig. 8A; Video 2), which implied the damages of the inner membrane within the mitochondria.

In the present study, we noticed that compared with the data in SNI group, Drp1 overexpression treatment significantly decreased mito (mitochondira) density within the SDH (SNI: 0.60 ± 0.08/μm^2^; SNI + OE: 0.42 ± 0.16/μm^2^; P = 0.0014) (Fig. 8B, D; Video 3). However, Drp1 OE treatment did not alter the mito perimeter (SNI: 1.25 ± 0.37 μm; SNI + OE: 1.16 ± 0.45 μm; P = 0.6769) (Fig. 8B, E; Video 3), the mito area (SNI: 0.12 ± 0.08 μm^2^; SNI + OE: 0.11 ± 0.08 μm^2^; P = 0.9154) (Fig. 8B, F; Video 3), or the mito circularity (SNI: 0.87 ± 0.07; SNI + OE: 0.90 ± 0.10; P = 0.2046) (Fig. 8B, G; Video 3). These results showed Drp1 up-regulation could reduce the number of mitochondria within the SDH but did not change the external appearance of the mitochondria.

Supplementary video related to this article can be found at https://doi.org/10.1016/j.redox.2021.102216

**Fig. 9:**
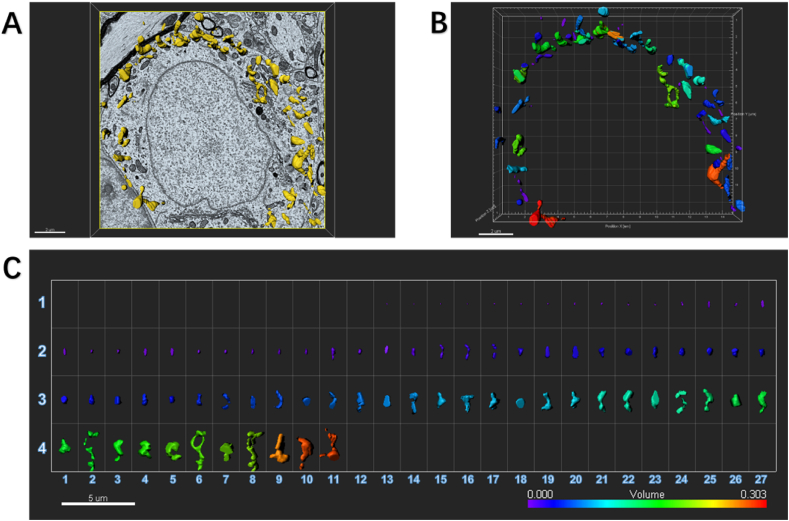
An example to show how an image of electron microscopy block captured from the ipsilateral SDH of SNI mice injected with Drp1 OE is processed for serial 3-dimensional reconstructions. SDH: spinal dorsal horn; SNI: spared nerve injury; OE: overexpression. (A) Imaris software was used to reconstruct SDH in the volume of 16 × 15.5 × 1.75 μm from 35 electron microscope images of continuous sections. Scale bar, 2 μm (B) The Vantage module of Imaris was used to establish three-dimensional reconstruction of mitochondria in the SDH of OE mice, and the plot type was presented in XYZ color format. Scale bar, 2 μm (C) Three-dimensional mitochondria were presented in Gallery format and arranged according to volume. Scale color was set according to the volume. In the Gallery format in the Drp1 OE group, there were 92 mitochondria captured from one neuronal soma in ipsilateral SDH. Among them, 68 mitochondria (74%) had small volume ranged from 0.001 μm^3^ to 0.050 μm^3^, 21 mitochondria (23%) had moderate volume ranged from 0.051 μm^3^ to 0.088 μm^3^, and only 3 mitochondria (3%) had large volume ranged from 0.089 μm^3^ to 0.127 μm^3^. Scale bar, 5 μm. (For interpretation of the references to color in this figure legend, the reader is referred to the Web version of this article.)

The following is the supplementary data related to this article:

Multimedia component 3 Imaris software was used to reconstruct mitochondria located in one neuronal cell within the SDH of SNI+Drp1 OE mouse. Electron microscope sections of the tissue were selected and 35 continuous electron microscope sections with a volume of 16 × 15.5 × 1.75 μm^3^ were reconstructed using Imaris software. About 92 mitochondria (yellow) in neurons were labeled with the surface creation. SNI: spared nerve injury; SDH: spinal dorsal horn; OE: overexpression. Related to Figure 8B and 9. Multimedia component 3

Then the effect of Drp1 OE on the internal structure of mitochondrial vacuole was analyzed according to the vacuole parameters by 3-dimension construction within the SDH. We could see Drp1 overexpression treatment did not alter vac (vacuole) density (SNI: 3.90 ± 1.45/mito; SNI + OE: 2.54 ± 1.22/mito; P = 0.2435) (Fig. 8B, H; Video 3), the vac perimeter (SNI: 0.27 ± 0.14 μm; SNI + OE: 0.28 ± 0.16 μm; P = 0.9859) (Fig. 8B, I; Video 3), or the vac area (SNI: 0.0036 ± 0.0048 μm^2^; SNI + OE: 0.0043 ± 0.0059 μm^2^; P = 0.9859) (Fig. 8B, J; Video 3). These results showed Drp1 up-regulation did not alter the internal vacuole characteristic within the SDH mitochondria.

By using serial 3-dimensional reconstruction analysis, in the Gallery format in the Drp1 OE group, there were fewer mitochondria (92) captured from one neuronal soma in ipsilateral SDH (Fig. 9), compared with those in SNI group. Among them, 68 mitochondria (74%) had small volume ranged from 0.001 μm^3^ to 0.050 μm^3^, 21 mitochondria (23%) had moderate volume ranged from 0.051 μm^3^ to 0.088 μm^3^, and 3 mitochondria (3%) had large volume ranged from 0.089 μm^3^ to 0.127 μm^3^. These results of serial 3-dimensional reconstruction analysis were consistent with the finding by electron microscopy that Drp1 up-regulation did not significantly change the external appearance of the mitochondria.

Compared with the data in SNI group, Drp1 down-regulation by RNAi significantly decreased mito (mitochondira) density within the SDH (SNI: 0.60 ± 0.08/μm^2^; SNI + RNAi: 0.29 ± 0.11/μm^2^; P < 0.0001) (Fig. 8C and D; Video 4), significantly increased the mito perimeter (SNI: 1.25 ± 0.37 μm; SNI + RNAi: 1.90 ± 0.82 μm; P < 0.0001) (Fig. 8C, E; Video 4), the mito area (SNI: 0.12 ± 0.08 μm^2^; SNI + RNAi: 0.28 ± 0.24 μm^2^; P < 0.0001) (Fig. 8C, F; Video 4), and the mito circularity (SNI: 0.87 ± 0.07; SNI + RNAi: 0.89 ± 0.11; P = 0.0399) (Fig. 8C, G; Video 4). These results showed Drp1 down-regulation could reduce the number of mitochondria within the SDH and change the external appearance of the mitochondria to be more swelling and more spheric.

Supplementary video related to this article can be found at https://doi.org/10.1016/j.redox.2021.102216

**Fig. 10:**
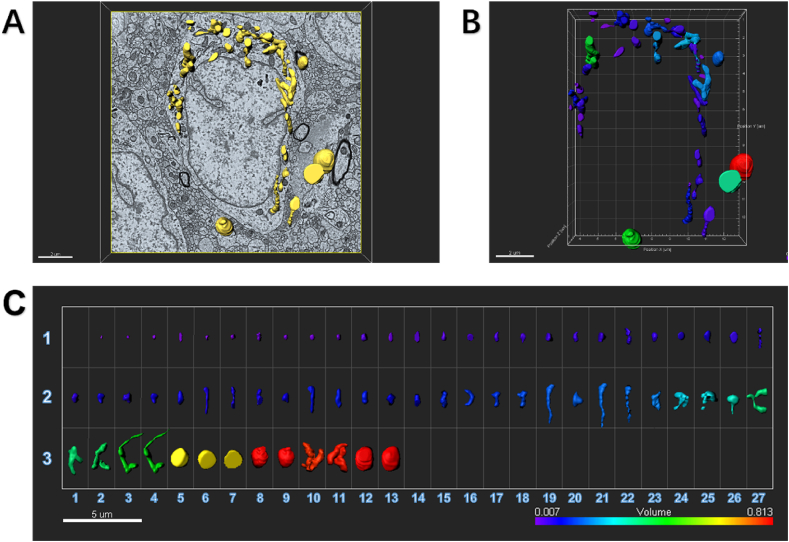
An example to show how an image of electron microscopy block captured from the ipsilateral SDH of SNI mice injected with Drp1 RNAi is processed for serial 3-dimensional reconstructions. SDH: spinal dorsal horn; SNI: spared nerve injury; RNAi: RNA interference. (A) Imaris software was used to reconstruct SDH in the volume of 16 × 15.5 × 1.75 μm from 35 electron microscope images of continuous sections. Scale bar, 2 μm (B) The Vantage module of Imaris was used to establish three-dimensional reconstruction of mitochondria in the SDH of RNAi mice, and the plot type was presented in XYZ color format. Scale bar, 2 μm (C) Three-dimensional mitochondria were presented in Gallery format and arranged according to volume. Scale color was set according to the volume. In the Gallery format in the RNAi group, there was remarkably reduced number of mitochondria (only 67) captured from one neuronal soma in ipsilateral SDH. We could see, in total 67 mitochondria, 50 mitochondria (75%) had small volume ranged from 0.001 μm^3^ to 0.050 μm^3^, 10 mitochondria (15%) had moderate volume ranged from 0.051 μm^3^ to 0.088 μm^3^, and 7 eight mitochondria (10%) had large volume ranged from 0.089 μm^3^ to 0.127 μm^3^. Scale bar, 5 μm. (For interpretation of the references to color in this figure legend, the reader is referred to the Web version of this article.)

The following is the supplementary data related to this article:

Multimedia component 4 Imaris software was used to reconstruct mitochondria located in one neuronal cell within the SDH of SNI+Drp1 RNAi mouse. Electron microscope sections of the tissue were selected and 35 continuous electron microscope sections with a volume of 16 × 15.5 × 1.75 μm^3^ were reconstructed using Imaris software. About 67 mitochondria (yellow) in neurons were labeled with the surface creation. SNI: spared nerve injury; SDH: spinal dorsal horn; RNAi: RNA interference. Related to Figure 8C and 10. Multimedia component 4

As for the internal structure of mitochondrial vacuole, Drp1 down-regulation significantly increased three key parameters, including vac density (SNI: 3.90 ± 1.45/mito; SNI + RNAi: 6.10 ± 3.29/mito; P = 0.0171) (Fig. 8C, H; Video 4), the vac perimeter (SNI: 0.27 ± 0.14 μm; SNI + RNAi: 0.53 ± 0.47 μm; P < 0.0001) (Fig. 8C, I; Video 4), and the vac area (SNI: 0.0036 ± 0.0048 μm^2^; SNI + RNAi: 0.0162 ± 0.0400 μm^2^; P = 0.0003) (Fig. 8C, J; Video 4).

By using serial 3-dimensional reconstruction analysis, in the Gallery format in the Drp1 RNAi group, there were much fewer mitochondria (67) captured (Fig. 10), compared with those in SNI group. Among them, 50 mitochondria (75%) had small volume, 10 mitochondria (15%) had moderate volume, and 7 mitochondria (10%) had large volume.

Drp1 RNAi increased the vacuole density to 1.56 fold, the vacuole perimeter to 1.97 fold and the vacuole area to 4.50 fold of those at SNI state, respectively. Due to the pivotal role of mitochondrial cristae in respiration process and energy conversion [54], the present electron microscope study of Drp1 RNAi inducing the SDH mitochondria changing in morphology from normal to be swollen, with broken mitochondrial cristae and more invalid vacuole compartments, implied Drp1 RNAi treatment could deteriorate mitochondrial damages induced by SNI.

### 2.7 Targeted up-regulation of Drp1 alleviated ROS elevation induced by SNI, while targeted down-regulation of Drp1 aggravated oxidative damage

Previous studies showed that level of reactive oxygen species (ROS) rose in neurons after pain and injury [41,42,45]. According to previous report [55], mitochondrial ROS levels were detected by DHE-ROS assay and fluorescence microscopy in the present study. SNI significantly increased ipsilateral level of ROS at 2 folds (SNI_ipsilateral_: 24.33 ± 1.50, P < 0.0001) in SDH when compared with that at contralateral side (SNI_contralateral_: 13.64 ± 1.73) (Supplementary Fig. 1), also when compared with that in control group at both contralateral side and ipsilateral side (Control_contralateral_: 12.33 ± 1.48, P < 0.0001; Control_ipsilateral_: 13.00 ± 1.14, P < 0.0001), which confirmed the previous reports [41,42,45]. Importantly, at the ipsilateral side, accompanied with the analgesic effect of targeted up-regulation of Drp1 within the SDH, the Drp1 OE treatment alleviated the increased level of SNI-induced ROS (SNI + OE_ipsilateral_: 13.23 ± 1.33, P < 0.0001) (Supplementary Fig. 1). At the contralateral side, the ROS level in SNI + OE group sustained at the level similar to that in SNI gruop (SNI + OE_contralateral_: 13.97 ± 1.21) (Supplementary Fig. 1). More importantly, consistent with the invalid effect of targeted down-regulation of Drp1 within the SDH, the Drp1 RNAi treatment further expanded ROS increase by SNI (SNI + RNAi_ipsilateral_: 27.70 ± 2.72, P = 0.0152) (Supplementary Fig. 1).

### 2.8 MitoQ and Mdivi-1 exerted analgesia effect on SNI-induced mechanical and thermal hyperalgesia

The mitochondrial-targeted antioxidant, mitoquinone (MitoQ), is able to modify mitochondrial function, and has analgesic effect on mechanical and thermal hyperalgesia in the NP induced by the chemotherapy drug vincristine (Vin) [36]. And previous studies have shown that the intrathecal treatment with mitochondrial division inhibitor-1 (Mdivi-1) could attenuate mechanical hyperalgesia [41,42]. But we were also aware of different voices. Zhang et al. [56] found that acute manipulation of Drp1 (mdivi-1) did not significantly change mitochondrial morphology, but played a causal role in maintaining mitochondrial respiration in adult cardiomyocytes by affecting endogenous Drp1, and played an important physiological role in cellular bioenergetics and ROS signal transduction. Bordt et al. [57] showed that mdivi-1 is not a specific Drp1 inhibitor, but acts by inhibiting mitochondrial complex I-dependent O_2_ consumption and reverses electron transfer-mediated production of ROS. What's more, their conclusions confirmed that mdivi-1 influences multiple aspects of mitochondrial function respiration and ROS even in the absence of Drp1, and it has limited utility in studies aiming to demonstrate a specific role for Drp1-dependent fission in biological processes. So it was indicated that the analgesic effect of MitoQ and Mdivi-1 was by restoring the effective mitochondrial respiratory function against oxidative stress. However, the effects of MitoQ and Mdivi-1 on SNI-induced mechanical and thermal hyperalgesia have not been elucidated.

One hour after intraperitoneal treatment of MitoQ at 14 days following SNI, we observed the mice exhibited the mechanical pain relief with decreased ipsilateral PWT when compared with the value prior to MitoQ injection (pre-MitoQ: 0.07 ± 0.00 g, post-MitoQ: 0.53 ± 0.12 g, P < 0.0001) (Supplementary Fig. 2A). MitoQ treatment also exhibited the thermal pain relief with decreased ipsilateral reaction latency when compared with the value prior to MitoQ injection (pre-MitoQ: 4.65 ± 0.39 g, post-MitoQ: 6.67 ± 1.71 g, P = 0.0207) (Supplementary Fig. 2A).

Similarly, 1 h after intraperitoneal treatment of Mdivi-1 at 14 days following SNI, the mice exhibited the mechanical pain relief with increased PWT when compared with the value prior to Mdivi-1 injection (pre-Mdivi-1: 0.08 ± 0.04 g, post-Mdivi-1: 0.32 ± 0.12 g, P = 0.0411) (Supplementary Fig. 2B). Mdivi-1 treatment also exhibited the thermal pain relief with longer reaction latency when compared with the value prior to injection (pre-Mdivi-1: 4.87 ± 1.28 s, post-Mdivi-1: 9.54 ± 2.82 s, P = 0.0041) (Supplementary Fig. 2B).

So it was firstly indicated there was the analgesic effect of MitoQ and Mdivi-1 on SNI-induced NP, likely by resisting against oxidative stress.

### 2.9 The analgesic effect of Drp1 up-regulation could persist at 28th day following SNI

In the section of 2.4.4, we presented the analgesic effect of up-regulation of Drp1 on pain hypersensitivity at 14th day following SNI surgery. Here we further confirmed the beneficial effect of Drp1 up-regulation on pain began significantly at 14th day, sustained till 21st day, and fad at 28th day following SNI (Supplementary Fig. 2C). In addition, the invalid effect of Drp1 down-regulation on pain persisted for 14 days, 21 days, and 28 days following SNI (Supplementary Fig. 2C). These results confirmed the targeted up-regulation of Drp1, instead of Drp1 down-regulation, alleviated pain symptoms in SNI mice.

## 3 Discussion

**Fig. 11:**
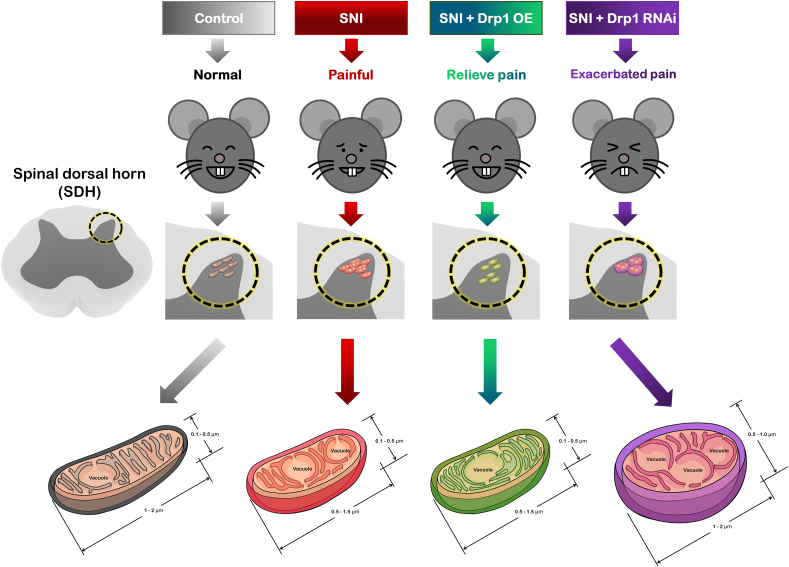
A schematic model proposing how Drp1, the key mitochondrial fission protein, modulates neuropathic pain and associated mitochondrial dysfunction. Pain stress (SNI) resulted in increased mitochondrial division, increased vacuoles and decreased volume of SDH mitochondria in the spinal dorsal horn (control, 0.1-0.5 × 1-2 μm; SNI, 0.1-0.5 × 0.5-1.5 μm), and the mitochondria tended to be round, suggesting that mitochondrial function be impaired. After Drp1 RNAi was used to down-regulate Drp1, mitochondria became larger (0.5-1.0 × 1-2 μm) and were accompanied by a large number of vacuoles, which further aggravated mitochondrial dysfunction. When Drp1 was up-regulated by Drp1 OE, the mitochondrial size returned to normal (0.1-0.5 × 1-2 μm), accompanied by the reduction of mitochondrial vacuoles, mitochondrial function was restored, and neuropathic pain was alleviated SNI: spared nerve injury; SDH: spinal dorsal horn; OE: overexpression; RNAi: RNA interference.

Based on the results of the current study, we proposed a model represented schematically in Fig. 11. Firstly, SNI mice showed painful and anxiety-like behaviors, which was associated with elevation of Drp1, as well as increased density of mitochondria within the pain control target, the spinal dorsal horn (SDH). Ultrastructural analysis further indicated the SDH mitochondria were sensitive to SNI stress by increasing their number, reducing their parameter and area, and transforming to be more spherical. Secondly, we showed the stable overexpression of Drp1 (Drp1 OE) alleviated SNI-induced pain behavior and abnormal gait. And, consistent with this, ultrastructural analysis indicated up-regulation Drp1 significantly improved the vacuole parameters including vac density, parameter and area, although Drp1 OE did not change mitochondrial density and their morphology. Thirdly, we showed the stable down-regulation by Drp1 RNA interference (RNAi) did not alleviate SNI-induced pain, and even deteriorated the SDH mitochondria to be more swollen and containing more vacuole structures. Our results supported that mitochondria were sensitive to NP and suggested Drp1 might be a novel therapeutic target for pain treatment.

### 3.1 Mitochondria in the nervous system are sensitive to NP

Our study strongly supports that mitochondria are sensitive to NP stress. The process of NP is the result of complex pathological changes caused by peripheral or central nerve injury [1,2]. Due to its complexity of pathogenesis and adverse reactions to drugs, its therapeutic treatment remains a challenge. Among all mechanistic studies, accumulating evidence has pointed to mitochondrial dysfunction [[34], [35], [36], [37], [38], [39], [40], [41], [42],^45^]. Neurons are dependent on mitochondria, which not only provide energy to power cellular function through oxidative phosphorylation, but also regulate cellular oxidation-reduction status, calcium levels, signal transduction, and apoptosis [58]. Various stress conditions are known to quickly trigger the disruption within mitochondria [59]. Bennett GJ et al., in 2014 [60] and Dai CQ et al., in 2020 [45] reviewed the extensive evidence which supports the hypothesis that mitochondrial dysfunction in nervous system is the fundamental causative factor in NP. Our results have supported that the mitochondria within the spinal dorsal horn (SDH), the key target in pain control, responded to NP stress by changing their structure and morphology.

### 3.2 Mitochondrial fission factor Drp1 was sensitive to NP

Our study also supports that mitochondrial fission factor Drp1 was sensitive to NP. In the nervous system, mitochondrial dynamics including fission and fusion play critical roles in maintaining functional mitochondria when neurons are exposed to metabolic or microenvironmental stresses [[16], [17], [18]]. The dynamin family of large GTPases regulates mitochondrial membrane dynamics in all eukaryotes: Drp1 for fission, and mitofusins (Mfn1/2) and optic atrophy-1 (Opa1) for fusion [[20], [21], [22], [23]]. Our own study has shown that Drp1 is distributed all over the whole nervous system, especially high at the spinal cord level [33]. In different NP model, Drp1 expression or its phosphorylated state is reported to either increase or decrease. It has been reported there was upregulation of Drp1 proteins at dorsal root ganglia (DRG) by immunohistochemical staining in the diabetic NP model [34], or by Western blot in the chronic constriction injury (CCI) NP model [39,40]. Xie M et al. have reported upregulation of Drp1 proteins or mRNAs at spinal cord by Western blot and RT-PCR in Complete Freund's adjuvant (CFA)-induced NP model [35]. Chen XJ et al. have reported upregulation of phosphorylated-Drp1 in primary cultured glial cells at spinal cord by Western blot in vincristine-induced NP model [36]. Li MY et al. also have observed the increased Drp1, but decreased phosphorylated-Drp1, at spinal cord by Western blot in bone cancer -induced NP model [38]. Similarly, Hao MM et al. have reported decreased phosphorylated-Drp1 at spinal cord by Western blot in bone cancer -induced NP model [37]. At the same time, Zhan L et al. have shown the increased Mfn1 and Opa1 proteins at DRG by Western blot CCI model [40]. Here we support Drp1 is sensitive to NP by the form of elevated expression, which might imply mitochondrial fission began to occur under the painful stress. But we did not perform the observation of phosphorylated Drp1 or activated Drp1 in other form, which should be studies in the next research.

### 3.3 Changes of mitochondrial morphology and vacuole ultrastructure following NP

Although many studies have deduced the mitochondrial dysfunction underlying NP, mainly based on the alternation of regulator factors involved in mitochondrial fission or fusion, such as Drp1, Mfn1, or Opa1, and so on, studying of mitochondria morphology in nervous system tissue is very limited. Our present studies are the first time to present very detailed description about mitochondrial changes in morphology and vacuole structure following SNI-induced NP. Seven important mitochondrial parameters were introduced, including: a, mitochondria (mito) density; b, mito perimeter; c, mito area; d, mito circularity; e, vacuole (vac) density; f, vac perimeter g, vac area (Fig. 2A). We have found SNI could induce mitochondria to be in more number, and at much shorter and smaller shape. We further observed the increased size of mitochondrial vacuoles following SNI. Our results are consistent with previous reports. Nieto et al. [61] have shown that mitochondrial alterations, including enlarged size (swelling) and vacuolization, were observed in axons of saphenous nerve in paclitaxel-induced NP model. Interestingly, Jin HW et al. have reported significantly increased incidence of swollen and vacuolated axonal mitochondria in A fibers and C fibers in paclitaxel-induced NP model [62].

We further believe the ultrastructural details are much more valuable than only focusing on the changes of expression level of Drp1. In the mitochondrial life cycle, Drp1 has been shown to induce distinct fates [63], of which one is the biogenesis of new mitochondria, the other is the clearance of dysfunctional mitochondria through mitophagy. Some studies have demonstrated ultrastructural damage to neurons. Some reports have revealed the increased number and decreased size of mitochondira in pyramidal neurons Alzheimer's disease (AD) brain [64,65]. And various Parkinson's disease (PD)-related neurotoxic molecules cause increased mitochondrial fission in vitro [66], suggesting that mitochondrial fusion and fission are involved in PD. More importantly, we have not only found SNI could induce more fragmented mitochondria within the SDH, but also observed the increased ROS, the byproduct of ATP synthesis, at the same time. So the mitochondrial dysfunction could be true in SNI. But further research is needed to detect whether or not these mitochondria exhibit decreased membrane potential [67,68] or the deficits of mitochondrial DNA (mtDNA) [59], which have been proved to be prominent features found in neuronal disorders.

### 3.4 ROS and NP

Reactive oxygen species (ROS) are generated by neurons, glia, and immune cells. Many previous studies have shown that ROS are involved in the hyperalgesia in NMDA-induced pain model [69] or capsaicin-induced pain model [70] at the spinal cord level, or in visceral pain model [71] at amygdala level, or in cisplatin-evoked [72] or CCI -evoked pain model [73] at DRG level, or in hypoxia/reoxygenation-evoked acute hyperalgesia [74] at microglia, or in spinal nerve ligation (SNL)-induced NP model at astrocytes [75]. Moreover, treatment of ROS donor t-BOOH could induce allodynia [76]. Previously Kallenborn-Gerhardt W [77] et al. and Grace PM et al. [78] have reviewed how the ROS is generated after NP enhances neuroexcitability in pain pathways and the potential therapeutic strategies for normalizing ROS. Our previous report [31] and the present results all indicate that increased ROS is generated at the spinal cord level following SNI pain.

Previous studies have shown that multiple ROS scavengers, such as phenyl-N-*tert*-butylnitrone (PBN), 4-hydroxy-2, 2, 6, 6-tetramethylpiperidin-1-oxyl (TEMPOL) or mito-TEMPO, or mitoquinone (MitoQ), could alleviate the enormous burden of pathological pain, including the analgesic effect of PBN on capsaicin-induced [79] or NMDA-induced hyperalgesia [70], TEMPOL on spinal cord injury-induced NP [75], or Mito-TEMPO on CCI-induced NP [40], or MitoQ on vincristine-induced NP [36]. MitoQ is a derivative of Coenzyme Q and is postulated to function as an anti-oxidant. Our present study is consistent with previous report [36] that MitoQ shows benefits in alleviating both mechanical and thermal hyperalgesia induced by SNI.

### 3.5 Targeted up-regulation of Drp1 attenuates SNI-induced pain

In 2011, Ferrari et al. reported that [41] intrathecal administration of ASO against Drp1 could markedly attenuate neuropathic mechanical hyperalgesia caused by HIV/AIDS antiretroviral and anticancer chemotherapy in rats. In 2016, Kanda et al. [42] reported that the intrathecal Drp1 ASO could markedly attenuate neuropathic mechanical hyperalgesia caused by gp120 in rats. Our present results are totally opposite to these two results. Three main different points should be taken into consideration. Firstly, the NP model is different. What we have found is that the targeted up-regulation of Drp1 by overexpression (OE) virus delivery within the SDH could alleviate SNI-induced mechanical and thermal pain symptoms. Secondly, the animal species is different. What we have used here is mouse. Thirdly, the regulation way of Drp1 is different. We have combined the up-regulation of Drp1 by AAV overexpressing (OE) Drp1 and the down-regulation of Drp1 by AAV expressing RNAi in the present study.

We think the mechanism underlying of the beneficial effect of Drp1 OE on pain could lie in its contribution to restoring the mitochondrial morphology, especially stabilizing inner mitochondrial membrane. It might be proven that Drp1 OE could alleviate the increased number of mitochondria and elevated level of ROS (Fig. 8, Fig. 9; Multimedia component 3, Multimedia component 4). Mitochondrial fission is an acute and adaptive response upon injury and stress [80]. Drp1 is very important for mitochondrial fission in nervous system. Drp1-deficient mice have shown severe abnormalities in neurons both at developmental stage and post-mitotic stage [81]. Accordingly, Drp1 could be a positive regulator of neuronal survival. So the increased Drp1 in highly polarized neurons ensures the rapid transport of mitochondria to sites of synaptic terminals, where local energy production is critical. Defects in mitochondrial transport can cause local energy depletion and disruption of Ca^2+^ buffering, which can ultimately trigger synaptic dysfunction and loss. So we think the mitochondrial fission is crucial in maintenance of gate control in pain stress. But the significance of mitochondrial fission in neurons following NP is still unclear.

### 3.6 Limitations

In the present study, we should admit there are at least two limitations. The first was that, unfortunately, Drp1 OE could not significantly up-regulate Drp1 mRNAs detected by qRT-PCR method, although Drp1 OE could significantly up-regulate Drp1 proteins detected by Western Blot. Secondly, it is well known that there are many neurons within SDH and the inhibitory GABAergic neurons releasing the inhibitory neurotransmitter gamma-aminobutyric acid (GABA) are assumed to play an important role in pain control. But our present observation did not distinguish the neuronal types. So in the future, it is necessary to use GABA associated transgenic mice to make research on mitochondrial plasticity.

In conclusion, we propose that the mitochondria at spinal cord level are sensitive to NP and the targeted up-regulation of mitochondrial fission factor Drp1 could attenuate pain hypersensitivity in SNI pain model. Despite challenges, progress in the understanding of the pathophysiology of mitochondria in NP is spurring the development of new diagnostic procedures and management for NP.

## 4 Methods

### 4.1 Animals

Adult male C57BL/6 J mice weighing 20 ± 2.5 g on average, were provided by the Experimental Animal Center of the Air Force Medical University, Xi'an, Shaanxi, China. The animals were randomly located under a 12-h light-dark cycle (8 a.m.–8 p.m.), with unrestricted access to food and water. All animal protocols were based on the procedures approved by the Animal Ethical and Welfare Committee for Institutional Animal Care and Use Committee (IACUC) of the Air Force Medical University (Permission NO. IACUC-20190107) and followed our institutional guidelines for the use of laboratory animals. All testings were done in a double-blinded manner.

### 4.2 Surgical procedures of SNI model

A model of SNI in mice was induced as described previously [6]. Mice were given pentobarbital anesthesia (50 mg/kg, i.p.) and an incision of approximately 1.5 cm was made on the superior border of the right hind limb. Then, the muscle was gently separated to reveal the main trunk of sciatic nerve and its 3 branches, which were bluntly separated. Spared nerve injury (SNI) was produced by ligating and severing a 2–4 mm segments of the common peroneal and tibia nerve (Fig. 1A). The sciatic nerve and its branches were only exposed in the control mice. After the operation, all mice were monitored for any signs of infection.

### 4.3 Behavioral assays

The experiments were done from 09:00 to 12:00 a.m. To minimize possible transfer influences and potential visual or olfactory effects, the mice were transferred to the testing room inside their home cages and allowed to acclimatize for at least 30 min. After each test, the surfaces of instrument were carefully cleaned with 75% alcohol to avoid interference with the scent of the previous mouse.

#### 4.3.1 Mechanical hypersensitivity

Baseline behavioral assessments were conducted within 2 weeks after SNI to identify the development of NP. Using von Frey (No.37450-275, Aesthesio, Italy) monofilaments, the paw withdrawal threshold (PWT) method was used to assess the hypersensitive response of mice to mechanical stimuli. Each von Frey filament was held perpendicular to the lateral aspect of the hind paw for approximately 5 s. The left hind paw was stimulated first, then the right hind paw (5 min apart). The mechanical threshold (in grams) is defined as the first filament that causes at least three withdrawals in five applications [49]. The PWT was measured using a series of calibrated von Frey filaments before SNI (day-1) and at post-operative day (SNI) 1, 2, 3, 5, 7, 14, 21 and 28.

#### 4.3.2 Thermal hyperalgesia

Reaction latencies to hot plate were measured pre- (baseline) and post-SNI to indicate thermal hyperalgesia [82]. Mice were placed on the hot plate (YLS-6B, Jinan, China) and the temperature was adjusted to 52 ± 0.5 °C. The time of the first nociceptive response (licking, flinching or jumping) of the surgical side of the paw was recorded and the animal was immediately removed from the hot plate. To avoid injury to the paw, the cut-off time was maintained at 25 s.

#### 4.3.3 Open field test (OFT)

The test was conducted using a 50 × 50 cm Plexiglas square box with 50 cm high walls as previously described [83]. Mice were placed in the middle of the Plexiglas arena and could freely explore the whole arena for 15 min [84].

#### 4.3.4 Elevated plus-maze (EPM)

Elevated plus-maze is made of Plexiglas and consists of 2 open arms (35 × 7 cm) and 2 opposite closed arms (35 × 7 cm), the latter surrounded by 15 cm high walls. The apparatus was raised to 50 cm above the ground. Each mouse was placed in the center of the apparatus, and allowed to move freely for 5 min [85]. Anxiety-like behavior was determined by measuring the preference of mice to open-arm for 5 min [86].

#### 4.3.5 CatWalk gait analysis

Gait analysis was performed using the CatWalk XT system (Noldus, the Netherlands), which has been proven a very reliable method for measuring pain-related behaviors [87]. The mouse was placed on the open end of an enclosed glass platform and walked across the glass floor voluntarily, during which a high-speed camera underneath the device captured images of each paw and transmitted the data to gait analysis software (CatWalk XT, version 10.6; Noldus) [46,88]. In this study, eight available parameters were identified to assess the dynamic behaviors relevant to NP [88,89]: (1) regularity index as the degree of inter-limb coordination as a percentage of complete coordination; (2) stand as the duration of a paw touching the glass plate; (3) body speed is calculated by counting the distance of the mouse's body (paws) from the initial contact with the glass plate to the next contact divided by the time required to move this distance; (4) standing on three as the percentage of time spent walking with three paws; (5) print length as the length of the paw print (horizontal direction); (6) print width as the width of the complete paw print (vertical direction); (7) max contact area as the maximum print area during paw contact; and (8) max contact mean intensity as the mean intensity at maximum paw contact.

### 4.4 Virus vector delivery

The recombinant adeno-associated viruses (rAAVs) expressing both EGFP and Drp1 were used to label and overexpress Drp1, which were produced by the Heyuan Biotechnology Co, Ltd (OBIO, Shanghai, China) at the following stock titers [in genome copies (GC)/ml)]: pAAV-CMV-MTS-Drp1-EGFP (enhanced green fluorescent protein) -3FLAG at 4.4 × 10^12^. The rAAVs that interfere with expression were produced by the Hanbio Biotechnology Co, Ltd (Shanghai, China) at the following stock titers [in genome copies (GC)/ml)]: pAAV-*m*-Drp1 shRNA-EGFP at 1.3 × 10^12^. Drp1 was labeled in the SDH of mice with EGFP-expressing rAAVs.

### 4.5 Virus injections

The AAV vector was injected into the SDH of mice as described previously [90]. Mice were anaesthetized with pentobarbital and the target vertebral column position was located using palpation. Remove the paraspinous muscle above the L4/5 vertebra and a partial laminectomy of the target side vertebral body was performed for injection. A microsyringe (RWD 79013, Shenzhen, China) was used to inject AAV with a target injection capacity of 300 nL and an injection speed of 50 nL/min. In order to inject the target into the SDH, stereotaxic apparatus (RWD 69100, Shenzhen, China) was used to move 500 nm to the side of the spinal cord, and the final depth of the tip was 300 nm. The needle was left in the spinal cord for another 10 min before it was removed. The wound was stratified with intermittent sutures and iodine disinfectant was applied to the closed wound.

### 4.6 Microscopic analysis

Virus-labeled Drp1 was observed in spinal cord histochemical staining sections, and co-immunostaining for DAPI (1:500; C1002, Beyotime Biotechnology, China) staining was conducted to mark the outline of sagittal spinal cord sections. The images were acquired on an Olympus IX73 (Japan)inverted microscope or an Olympus FV1200(Japan)confocal microscope, and the images obtained were processed using the open source FIJI (NIH ImageJ, USA) software (http://fiji.sc/Fiji).

### 4.7 Western blot

Western blot experiments were performed as previously described [91]. Briefly, mouse spinal cord neuron cell samples were homogenized and processed for Western blot analysis 4 w after viral transfection, and the lysates from at least three spine cord tissues per group were clarified by centrifugation at 12,000 rpm for 10 min. The tissues were lysed in radioimmunoprecipitation assay (RIPA) buffer (No. 2020818, Solarbio, Beijing, China) containing protease/phosphatase inhibitor cocktail (Sigma, Germany). NanoDrop™ One Microvolume UV–Vis Spectrophotometer (Thermo Scientific™ 840274100, USA) was used to measure the protein concentration of RIPA lysates. Samples (20 mg) loaded on 12.5% acrylamide gel (PG111, EpiZyme, China) and blotted onto methanol activated PVDF membranes (IPVH00010, Millipore, USA). Immunoblots were soaked in 5% skimmed milk for 2 h at room temperature and subsequently detected with the following primary antibodies (4 °C, overnight): rabbit-*anti*-Drp1 (1:250, ab184247, Abcam, CA), rabbit-*anti*-Mfn1 (1:1000, #14739, CST, CA), rabbit-*anti*-Mfn2 (1:1000, #9482, CST, CA), mouse-*anti*-β-actin (1:5000, A01010, Abbkine, China) and rabbit-*anti*-β-actin (1:5000, A01011, Abbkine, China). Then incubated with the corresponding horseradish peroxidase (HRP)-conjugated secondary antibodies (1:5000, A21010, mouse; 21020, Rabbit, Abbkine, China). The bands were detected with enhanced chemiluminescence (SQ202L, EpiZyme, China) followed by exposure to luminometer (Fusion FX6-XT, Vilber, USA). The open source FIJI (NIH ImageJ) software was used to quantify the intensity of the immunoreactive bands of interest on autoradiograms. Target protein levels were normalized relative to β-actin levels and expressed as a fold change relative to the original group.

### 4.8 Real time-quantitative PCR (qRT-PCR)

To measure the changes of mitochondrial genes expression levels, the mice were sacrificed for qRT-PCR measurement. Spinal cords were collected and homogenized, and then Trizol reagent (Invitrogen, CA) was used to measure total RNA. The extracted RNA was used as a template to synthesize the corresponding cDNA and to perform qRT-PCR assays. The genes detected included the mitochondrial morphology-related genes of Drp1 (Tsingke, Beijing, China), Mfn1 (Tsingke, Beijing, China) and Mfn2 (Tsingke, Beijing, China). The internal reference is β-actin (Tsingke, Beijing, China). The primer sequences are shown in Table 1. The reaction conditions: pre-denaturation 95 °C, 15 s, followed by denaturation 95 °C, 5 s for each step, annealing, extension 60 °C for 30 s for 40 cycles. After amplification, the homogeneity of the product was checked using a lysis curve. The relative content was statistically analyzed using the 2^-△△ Ct^ method.

**Table 1:** Sequences of the primers employed for amplification of mRNAs encoding Drp1, Mfn1, Mfn2 and β-actin by qRT-PCR.

mRNA	Sequences (5′-3’)	
Drp1	Forward: Reverse:	5′-AACAGGCAACTGGAGAGGAA-3′ 5′-GCAACTGGAACTGGCACAT-3′
Mfn1	Forward: Reverse:	5′-GGTCTGCTTTCCTGCTCTCT-3′ 5′-CTTTCTGCTCCCATTTCACC-3′
Mfn2	Forward: Reverse:	5′-CCTGGGATCGATGTTACCAC-3′ 5′-AACTGCTTCTCCGTCTGCAT-3′
β-actin	Forward: Reverse:	5′-AACAGTCCGCCTAGAAGCAC-3′ 5′-CGTTGACATCCGTAAAGACC-3′

### 4.9 Transmission electron microscopy (TEM)

Mice were anaesthetized and perfused sequentially with 0.01 M PBS and 4% formaldehyde with 0.1% glutaraldehyde in PBS. Then, the spinal cord tissues were placed into 4% glutaraldehyde solution for tissue fixation. After fixation, each spinal cord tissue was washed twice with 0.1 M PB. This was followed by 1% osmic acid (TED PELLA, Inc. No. 18451) for staining for 2 h. Then, 50%, 70%, and 90% ethanol and 100% acetone were used respectively for dehydration, followed by acetone: embedding agent (Embed 812, DDSA, NMA, DMP-30) = 1:1 for 2 h at room temperature. Lastly, the tissue was removed from the embedding agent and stored overnight at room temperature. The following day, the tissue was placed into a special electron microscope plate and incubated at 60 °C for 48 h. A 70 nm thick section was prepared using an ultra-thin sectioning machine. The slices were placed on a copper grid and stained with lead nitrate and uranyl acetate for 10 min each. Images were recorded using a TEM (JEM 1400, Olympus, Japan). The mitochondrial morphology data were analyzed applying ImageJ-based image analysis. Finally, 30 consecutive tissue sections were selected for the TEM analysis and sections of about 1500 nm thickness were obtained.

### 4.10 Morphometric analysis

The size of each mitochondria was obtained by drawing the electron microscopy sagittal profile of the mitochondria in SDH. The scale was set according to the bar of the picture in ImageJ. By using the freehand selections of this software, the outline of mitochondria was carefully drawn, and then the perimeter and area of the mitochondria were measured. The circularity of mitochondrial was determined according to the method previously described [32], that is, circularity = 4πarea/perimeter^2^. Hand counted the number of mitochondria in the SDH in each image to get the total number of mitochondria, and then calculated the density of the mitochondria. The same methods were used to calculate the area, perimeter and density of the vacuoles in the mitochondria.

Three-dimensional reconstruction of mitochondria: 30 images in tif format were converted to Imarisfiles (.ims) using (ImarisFileConverter X64, Version 9.0.1, BitPlane, Switzerland). 3D reconstruction and subsequent analysis was done using Imaris software (BitPlane, Switzerland). All mitochondria surface tracings were based on the contour tracing tool and carried out manually/semi-automatically. For surface creation, a total of 35 consecutive ultrathin sections (50 nm in thickness) were traced and image data were collected by electron microscopy. The surface at maximum resolution was created to ensure that the tracing lines match the surface boundaries. This allows the 3D reconstruction of the object from 2D images.

### 4.11 ROS assay

To detect ROS, spinal cord tissues were promptly frozen and cut into 15-μm-thick slices at −20 °C and then adhered to glass slides. The following steps were strictly in line with the standard protocol of manufacturer's instructions (BB-470513, BestBio, China) [55]. Briefly, the spinal cord samples were stained with ROS probe (DHE) for 30 min at 37 °C in darkness. DHE was oxidized to be ethidium oxide by intracellular ROS after permeating the cell membrane. Then DAPI (1:500; C1002, Beyotime Biotechnology, China) was applied for 30 s to sketch the outline of spinal cord. Fluorescence was detected using an Olympus IX73 inverted fluorescence microscope (Olympus, Japan). The images were observed at 610 nm wavelength.

### 4.12 Drug treatment

MitoQ (MCE, HY-100116 A, USA; 50 mg/kg) [92] and Mdivi-1 (MCE, HY-15886, USA; 10 mg/kg) [93] were intraperitoneally injected into the mice of SNI-induced neuropathic pain two weeks later, respectively. So did the Drp1 OE and Drp1 RNAi group. And 1 h later, all three groups of mice were tested for mechanical and thermal pain by using von Frey and the hot plate, respectively.

### 4.13 Statistical analysis

The statistical comparison methodologies for each study method are given in the figure legends. Quantitative data are expressed as mean ± SD. One-way ANOVA was used to assess differences between groups. Differences between groups were evaluated by one-way analysis of variance, and the Turkish post hoc test was used for multiple comparisons between groups. Statistical analyses were performed using SPSS 15.0. p < 0.05 was considered a statistically significant difference.

## Authors' contributions

K.L.Z., S.J.L. and R.Q.W. conducted Western blot and qRT-PCR. K.L.Z., S.J.L. and H.L. conducted stereotaxic surgery and behavioral test. B.Z.L., H.L. and K.F.L. performed animal preparation. K.L.Z., F.F.W., Z.L. and B.Z.L. analyzed data. Y.Y.W., H.Y. and Y.L.Y. designed studies. K.L.Z. and X.Y.P. wrote the draft manuscript. S.J.L. and X.Y.P. plotted the three-dimensional reconstruction of mitochondria. X.Y.P., K.L.Z. and Y.Y.W. draw pattern diagrams and layout. N.S.Q., Y.Y.W. and H.Y. supervised the experiments and revised the manuscript. All the authors read and approved the final manuscript.

## Conflicts of interest

The authors have declared that no conflict of interest exists.

## Appendix A Supplementary data

Multimedia component 9 Multimedia component 9

figs1 Targeted up-regulation of Drp1 alleviated ROS elevation induced by SNI, while targeted down-regulation of Drp1 aggravated oxidative damage. (A) Immunofluorescence results of confocal imaging to detect ROS expression homeostasis in all four groups. Targeted regulation of Drp1AAV virus (green) and ROS (red). Scale bars = 500 μm. (B) ROS levels defined as ROS area/total area according to the staining results (n = 6 for each group). Two-tailed unpaired separate variance estimation *t*-test. Data are presented as mean ± S.D. ROS: reactive oxygen species; AAV: adeno-associated virus; SNI: spared nerve injury; OE: overexpression; RNAi: RNA interference. figs1

figs2 MitoQ and Mdivi-1 exerted analgesia effect on SNI-induced mechanical and thermal hyperalgesia; and the analgesic effect of Drp1 up-regulation could persist at 28th day following SNI. (A, B) The PWT of von Frey and reaction latencies to hot plate test showed that drug intervention alleviated ipsilateral mechanical sensitivity and thermal hyperalgesia in SNI-aggravated C57BL/6 mice (n = 6 for each group). (C) The PWT tested by von Frey showing overexpression of Drp1 alleviated ipsilateral pain-induced mechanical sensitivity and thermal hyperalgesia in SNI mice, and the effect lasted at least 3 weeks (n = 6 for each group). Data are presented as mean±S.D. See Supplemental Table 3-5 for detailed information. Kruskal-Wallis *H* test with Nemenyi multiple comparisons test. SNI: spared nerve injury; MitoQ: Mitoquinone; OE: overexpression; RNAi: RNA interference. figs2
