# Supplementary material for: Targeted up-regulation of Drp1 in dorsal horn attenuates neuropathic pain hypersensitivity by increasing mitochondrial fission
Source: Redox Biol. 2021 Dec 20;49:102216. doi: 10.1016/j.redox.2021.102216 (PMC8718665; doi:10.1016/j.redox.2021.102216)
Supplement: Multimedia component 5 [file mmc5.pdf]

Supplemental Table 1. Results of mechanical hypersensitivity (von Frey test, g)

Group		Day-1		Day1		Day2		Day3		Day5		Day7		Day14	
		R	L	R	L	R	L	R	L	R	L	R	L	R	L
Control	1	0.60	1.00	1.00	1.00	0.40	0.40	0.60	0.60	0.40	0.60	1.00	0.60	1.00	0.60
	2	1.00	1.00	1.00	1.00	0.60	0.60	0.40	0.60	0.60	0.60	1.40	1.00	1.00	1.00
	3	1.40	1.40	1.40	1.00	1.00	0.40	0.60	0.60	0.60	1.00	1.40	1.00	1.40	1.00
	4	1.00	1.00	0.60	1.00	0.60	0.40	0.60	0.60	0.60	0.60	0.60	0.60	1.00	1.00
	5	1.00	1.00	0.60	0.60	0.60	0.60	0.40	0.60	1.00	0.60	1.40	0.60	1.40	0.60
	6	1.00	1.00	1.00	1.00	0.60	0.60	0.60	0.60	1.00	0.60	1.00	1.00	1.00	1.00
Sham	1	1.00	1.00	1.00	0.60	0.60	0.60	0.60	0.60	1.00	0.60	0.60	0.60	1.00	0.60
	2	1.40	1.00	1.40	0.60	0.60	0.60	0.60	0.40	1.00	0.60	1.00	0.40	1.00	0.60
	3	1.00	1.00	1.00	0.60	1.00	0.40	0.60	0.60	1.00	0.60	1.00	0.60	1.00	0.60
	4	1.40	1.40	1.40	0.60	0.60	0.60	0.60	0.60	0.60	0.60	1.00	0.60	1.00	1.00
	5	0.60	1.00	1.00	1.00	0.40	0.60	0.60	0.40	1.00	1.00	1.00	0.60	0.60	0.60
	6	1.00	1.00	1.00	0.60	0.60	0.60	0.60	0.60	1.00	0.60	1.00	0.60	0.60	0.60
SNI	1	1.00	1.00	0.60	0.40	0.40	0.16	0.40	0.16	0.16	0.16	0.40	0.40	1.00	0.07
	2	1.00	1.40	1.00	0.40	0.40	0.16	0.60	0.16	0.40	0.16	0.40	0.16	1.00	0.07
	3	1.00	1.00	0.60	0.40	0.60	0.16	0.60	0.16	0.40	0.16	0.16	0.16	0.60	0.04
	4	1.40	1.40	0.60	0.40	0.60	0.40	0.60	0.40	0.40	0.40	0.40	0.16	0.40	0.07
	5	1.00	1.00	0.60	0.40	0.60	0.16	0.40	0.16	0.40	0.40	0.16	0.16	0.60	0.07
	6	0.60	0.60	0.60	0.16	0.40	0.40	0.60	0.40	0.40	0.16	0.40	0.16	1.00	0.04
SNI+Drp1 OE	1	1.00	1.00	0.40	0.40	0.40	0.40	0.40	0.40	0.40	0.40	0.60	0.40	1.00	0.60
	2	0.60	1.40	0.40	0.16	0.60	0.16	0.40	0.40	0.40	0.16	0.40	0.16	0.60	0.60
	3	1.00	0.60	0.60	0.07	0.40	0.16	0.40	0.16	0.40	0.16	0.40	0.16	1.00	0.60
	4	0.60	1.00	0.40	0.16	0.40	0.07	0.40	0.16	0.40	0.40	0.40	0.40	0.60	0.40
	5	1.00	1.00	0.40	0.16	0.40	0.16	0.40	0.16	0.40	0.16	0.40	0.16	1.00	0.40
	6	0.60	0.60	0.60	0.40	0.40	0.40	0.40	0.40	0.40	0.16	0.40	0.40	1.00	1.00
	1	1.00	1.00	0.16	0.04	0.40	0.04	0.40	0.04	0.16	0.07	0.16	0.07	0.60	0.16

**SNI+Drp1 RNAi**	**2**	1.00	1.00	0.40	0.16	0.40	0.16	0.16	0.04	0.16	0.04	0.16	0.07	0.40	0.16
	**3**	1.00	0.60	0.40	0.16	0.40	0.16	0.16	0.16	0.40	0.16	0.40	0.16	0.60	0.16
	**4**	1.00	1.00	0.16	0.04	0.16	0.04	0.16	0.16	0.16	0.16	0.40	0.16	0.60	0.16
	**5**	1.00	1.40	0.16	0.04	0.40	0.04	0.16	0.16	0.40	0.16	0.40	0.07	0.40	0.16
	**6**	1.00	1.00	0.40	0.04	0.40	0.16	0.16	0.16	0.16	0.07	0.16	0.07	0.40	0.07

L = left (lpsilateral), R = right (contralateral).
