# Supplementary material for: Targeted up-regulation of Drp1 in dorsal horn attenuates neuropathic pain hypersensitivity by increasing mitochondrial fission
Source: Redox Biol. 2021 Dec 20;49:102216. doi: 10.1016/j.redox.2021.102216 (PMC8718665; doi:10.1016/j.redox.2021.102216)
Supplement: Multimedia component 6 [file mmc6.pdf]

Supplemental Table 2.
Results of thermal hyperalgesia (Hot plate test, s)

Group		Day-1	Day1	Day2	Day3	Day5	Day7	Day14
Control	1	11.11	10.21	10.81	10.60	10.75	10.72	10.46
	2	10.26	9.66	11.16	10.99	10.06	10.44	9.99
	3	10.31	10.21	10.65	10.42	9.88	11.22	10.56
	4	9.74	9.77	10.62	10.26	10.86	10.45	10.92
	5	10.08	10.26	10.66	10.89	10.50	10.50	10.88
	6	10.08	11.12	10.63	10.60	10.65	10.67	10.40
Sham	1	11.26	9.61	10.19	10.44	10.76	10.22	9.86
	2	10.50	9.78	9.72	8.75	11.01	10.32	10.36
	3	9.98	8.69	9.95	9.07	10.56	10.66	10.06
	4	10.17	9.15	9.48	9.85	10.88	10.76	9.83
	5	10.33	9.36	9.97	9.27	11.02	10.20	9.98
	6	10.17	8.95	9.82	9.89	10.35	10.28	10.31
SNI	1	10.62	5.24	4.96	3.46	4.90	4.66	5.12
	2	9.74	6.02	5.20	3.02	5.34	5.60	4.71
	3	10.13	5.31	5.34	3.45	5.83	5.55	5.25
	4	10.65	5.90	5.32	2.81	6.00	5.43	4.75
	5	10.71	5.02	5.23	3.60	5.37	5.40	4.89
	6	9.73	6.21	4.59	3.43	5.90	4.87	5.20
SNI+Drp1 OE	1	10.29	5.14	4.65	7.58	9.23	8.16	8.89
	2	11.32	5.30	5.02	6.65	8.29	8.04	8.61
	3	10.21	4.88	4.68	7.02	7.83	7.90	6.84
	4	9.52	4.56	5.21	7.19	7.61	7.37	8.60
	5	11.40	4.07	5.53	7.60	8.28	8.56	8.96
	6	11.03	4.73	4.83	7.02	7.50	7.22	8.61
SNI+Drp1 RNAi	1	11.47	3.70	4.87	4.54	3.31	3.55	3.73
	2	11.61	4.73	4.93	4.95	4.71	5.50	4.86
	3	10.41	4.92	4.73	5.62	6.05	5.20	5.99
	4	9.80	5.55	5.22	4.54	5.82	5.09	5.92
	5	10.98	6.73	5.20	3.62	5.04	4.12	4.17
	6	12.15	4.37	4.67	3.38	3.91	3.09	3.74
