# Supplementary material for: Targeted up-regulation of Drp1 in dorsal horn attenuates neuropathic pain hypersensitivity by increasing mitochondrial fission
Source: Redox Biol. 2021 Dec 20;49:102216. doi: 10.1016/j.redox.2021.102216 (PMC8718665; doi:10.1016/j.redox.2021.102216)
Supplement: Multimedia component 7 [file mmc7.pdf]

Supplemental Table 3.

Results of mechanical hypersensitivity (von Frey test, g)

Group		Pre-drug		Post-drug	
		R	L	R	L
Mdivi-1	1	0.40	0.07	0.40	0.16
	2	0.60	0.16	0.40	0.40
	3	1.00	0.07	0.60	0.40
	4	0.60	0.07	0.60	0.40
	5	0.40	0.07	0.40	0.16
	6	0.60	0.04	1.00	0.40
MitoQ	1	0.60	0.07	0.60	0.60
	2	0.60	0.07	0.60	0.60
	3	0.60	0.07	0.60	0.40
	4	0.60	0.07	0.60	0.60
	5	0.60	0.07	0.60	0.40
	6	0.60	0.07	0.60	0.60

L=left (lpsilateral), R=right (contralateral).
