# Supplementary material for: Targeted up-regulation of Drp1 in dorsal horn attenuates neuropathic pain hypersensitivity by increasing mitochondrial fission
Source: Redox Biol. 2021 Dec 20;49:102216. doi: 10.1016/j.redox.2021.102216 (PMC8718665; doi:10.1016/j.redox.2021.102216)
Supplement: Multimedia component 8 [file mmc8.pdf]

Supplemental Table 4.

Results of thermal hyperalgesia (Hot plate test, s)

Group		Pre-drug	Post-drug
Mdivi-1	**1**	7.01	12.95
	**2**	5.13	12.57
	**3**	4.48	8.48
	**4**	3.33	5.64
	**5**	5.30	9.61
	**6**	3.99	7.97
MitoQ	**1**	4.49	6.91
	**2**	4.64	5.53
	**3**	5.01	8.61
	**4**	5.00	8.64
	**5**	4.80	5.88
	**6**	3.97	4.43
