# Supplementary figures and images for: Targeted up-regulation of Drp1 in dorsal horn attenuates neuropathic pain hypersensitivity by increasing mitochondrial fission
Source: Redox Biol. 2021 Dec 20;49:102216. doi: 10.1016/j.redox.2021.102216 (PMC8718665; doi:10.1016/j.redox.2021.102216)

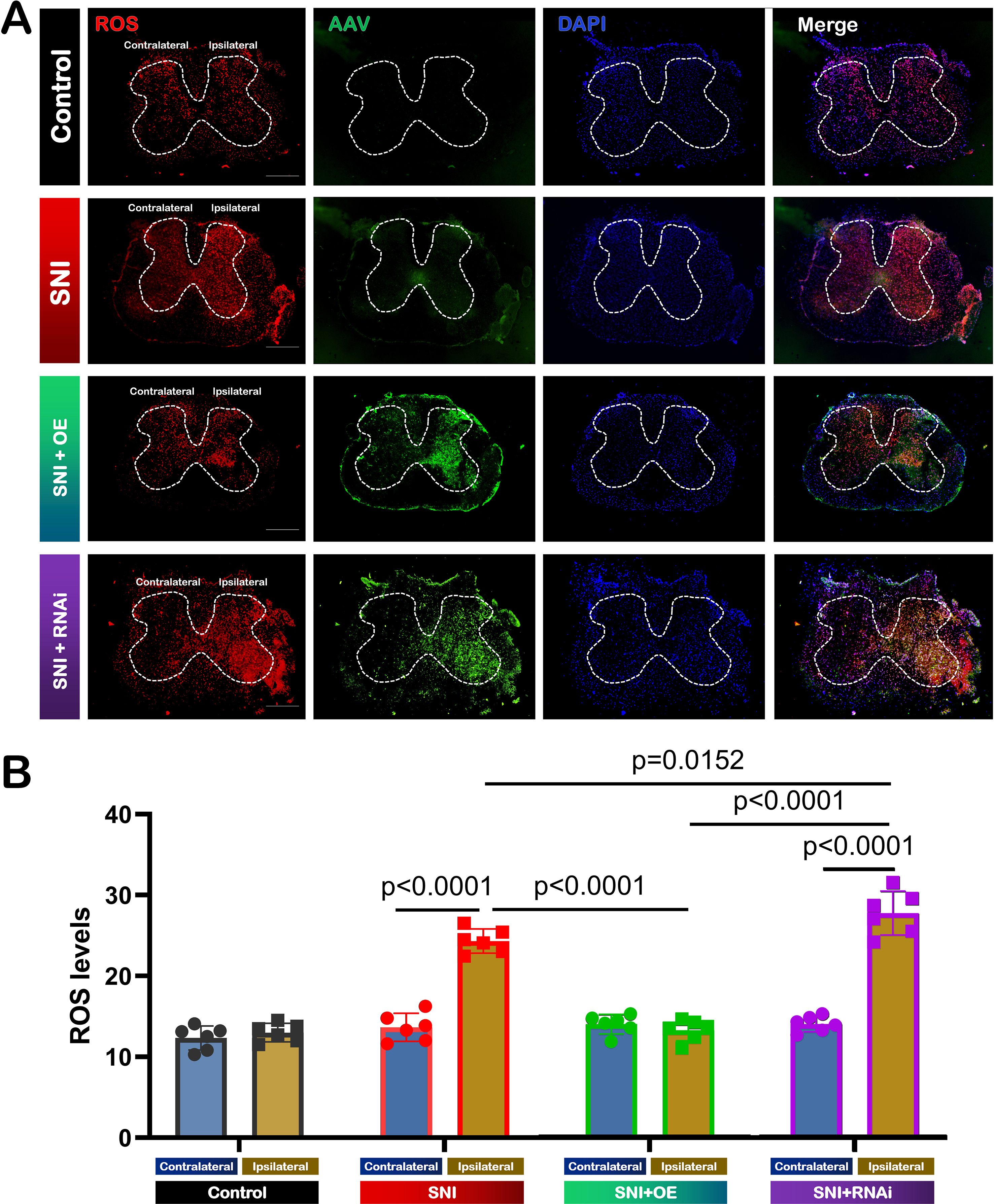

Supplement: figs1 — Targeted up-regulation of Drp1 alleviated ROS elevation induced by SNI, while targeted down-regulation of Drp1 aggravated oxidative damage. (A) Immunofluorescence results of confocal imaging to detect ROS expression homeostasis in all four groups. Targeted regulation of Drp1AAV virus (green) and ROS (red). Scale bars = 500 μm. (B) ROS levels defined as ROS area/total area according to the staining results (n = 6 for each group). Two-tailed unpaired separate variance estimation t-test. Data are presented as mean ± S.D. ROS: reactive oxygen species; AAV: adeno-associated virus; SNI: spared nerve injury; OE: overexpression; RNAi: RNA interference. [file mmcfigs1.jpg]

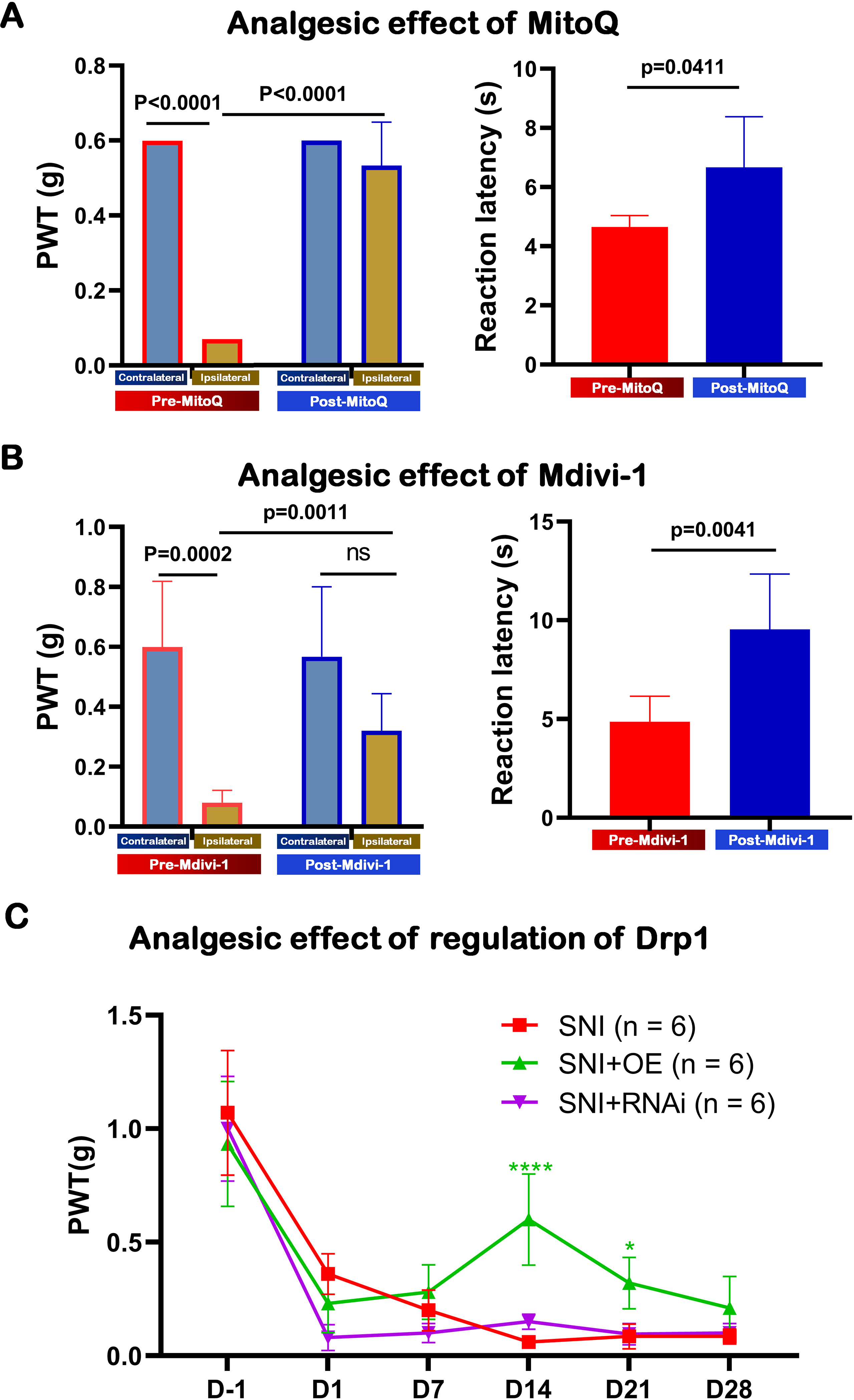

Supplement: figs2 — MitoQ and Mdivi-1 exerted analgesia effect on SNI-induced mechanical and thermal hyperalgesia; and the analgesic effect of Drp1 up-regulation could persist at 28th day following SNI. (A, B) The PWT of von Frey and reaction latencies to hot plate test showed that drug intervention alleviated ipsilateral mechanical sensitivity and thermal hyperalgesia in SNI-aggravated C57BL/6 mice (n = 6 for each group). (C) The PWT tested by von Frey showing overexpression of Drp1 alleviated ipsilateral pain-induced mechanical sensitivity and thermal hyperalgesia in SNI mice, and the effect lasted at least 3 weeks (n = 6 for each group). Data are presented as mean±S.D. See Supplemental Table 3-5 for detailed information. Kruskal-Wallis H test with Nemenyi multiple comparisons test. SNI: spared nerve injury; MitoQ: Mitoquinone; OE: overexpression; RNAi: RNA interference. [file mmcfigs2.jpg]
